# Supplementary material for: Progressive active mobilization with dose control and training load in critically ill patients (PROMOB): Protocol for a randomized controlled trial
Source: PLoS One. 2020 Sep 3;15(9):e0238352. doi: 10.1371/journal.pone.0238352 (PMC7470388; doi:10.1371/journal.pone.0238352)
Supplement: S1 File — (DOCX) [file pone.0238352.s002.docx]

| **Intervention Procedures** | | | | |
| --- | --- | --- | --- | --- |
| Usual care group (UCG): Receive mobilization according to the routine of the service: passive kinesiotherapy for patients with low level of consciousness, unable to cooperate and perform voluntary active movements; active exercises and mobility training for cooperative patients and capable of performing active movements voluntarily; eventually use of electrostimulation and passive orthostatism. | | | | |
| *Intervention Group (IG): Patients will undergo a protocol of progressive mobilization with individualized dose control and training load stratified according to functional levels and performance. | | | | |
| **Functional levels** | | | | |
| **Levels** | **Initial training** | **Training volume increase** | **Equipment** | **Repetitions** |
| N1 | Bridge and Rolling for both sides | Maximal triple flexion / extension of lower limbs | none | highest number you can get in 20 seconds |
| N2 | Transfer from supine to sit on both sides | Exercise of trunk rotation and anterior and posterior reach of a ball. | ball |  |
| N3 | transfer from sit to stand | Rit up from a chair throwing the ball on the wall | ball |  |
| N4 | Walking | Crouch / lift throwing ball on the wall | ball |  |
| **Frequency of protocol:** *The protocol will be applied once a day, 5 times a week; 8 series of each movement, alternating 20 seconds of execution with 10 seconds of rest. | | | | |
| **Varable/outcome** | **Hypothesis** | **Measure** | **Method of analysis** | |
| Muscle mass | The protocol can prevent the loss of muscle mass. | Cross-sectional area and muscle thickness | ultrasound, ultrasonography using an 8 MHz 5.6 cm linear transducer array | |
| Muscle strength | The protocol can prevent the loss of muscle strength. | MRC sum-score, Handgrip, Hand held and Five Times Sit to Stand Test | immediately after randomization, after 3 days and at intervals of 7 to 28th day or discharge from ICU | |
| ICUAW | The protocol can prevent the ICUAW. | MRC sum-score | score lower than 48 | |
| Functional Status | The protocol can prevent the functional decline | FSS, SOMS and Timed up-and-go score | at 28 days after randomization or discharge from ICU | |
| Hospitalization days, days with mechanical ventilation and Mortality | Reduces hospitalization days, days with mechanical ventilation and Mortality | Days Mortality | at 28 days after randomization or discharge from ICU | |

Summary study chart
