# Supplementary material for: Progressive active mobilization with dose control and training load in critically ill patients (PROMOB): Protocol for a randomized controlled trial
Source: PLoS One. 2020 Sep 3;15(9):e0238352. doi: 10.1371/journal.pone.0238352 (PMC7470388; doi:10.1371/journal.pone.0238352)
Supplement: S2 File — (DOCX) [file pone.0238352.s003.docx]

**The TIDieR (Template for Intervention Description and Replication) Checklist*:**


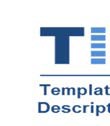

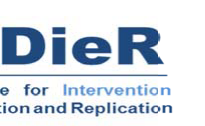


Information to include when describing an intervention and the location of the information

m

| **PROGRESSIVE ACTIVE MOBILIZATION WITH DOSE CONTROL AND TRAINING LOAD IN CRITICALLY ILL PATIENTS (PROMOB): PROTOCOL FOR A RANDOMIZED CONTROLLED TRIAL** | Primary paper (page or appendix number) | |  |
| --- | --- | --- | --- |
| **BRIEF NAME** |  | |  |
| **1.** Provide the name or a phrase that describes theintervention. | Cover page; Line 1 | |  |
| **WHY** |  | |  |
| **2.** Describe any rationale, theory, or goal of the elements essential to theintervention. | Introducion: Line 54-75 | |  |
| **WHAT** |  | |  |
| **3.** Materials: Describe any physical or informational materials used in the intervention, includingthose  provided to participants or used in intervention delivery or in training of intervention providers.  Provide information on where the materials can be accessed (e.g. online appendix, URL). | Methods: Line 130-202 | |  |
| **4.** Procedures: Describe each of the procedures, activities, and/or processes used in theintervention,  including any enabling or support activities. |  |  |  |
| **WHO PROVIDED** |  | |  |
| **5.** For each category of intervention provider (e.g. psychologist, nursing assistant), describetheir  expertise, background and any specific training given. | Methods: Line 141-143 | |  |
| **HOW** |  | |  |
| **6.** Describe the modes of delivery (e.g. face-to-face or by some other mechanism, such as internetor  telephone) of the intervention and whether it was provided individually or in a group. | Methods: Line 137 and 140 | |  |
| **WHERE** |  | |  |
| **7.** Describe the type(s) of location(s) where the intervention occurred, including anynecessary  infrastructure or relevant features. | Methods: Line 92-93 | |  |
| **WHEN and HOW MUCH** | | |  |
| **8.** Describe the number of times the intervention was delivered and over what period of timeincluding  the number of sessions, their schedule, and their duration, intensity or dose. | | | Methods: Line 161-186 |
| **TAILORING** | | |  |
| **9.** If the intervention was planned to be personalised, titrated or adapted, then describe what,why,  when, and how. | | | Methods: Line 168-177 |
| **MODIFICATIONS** | | |  |
| **10.ǂ** If the intervention was modified during the course of the study, describe the changes (what,why,  when, and how). | | | N/A |
| **HOW WELL** | | |  |
| **11.** Planned: If intervention adherence or fidelity was assessed, describe how and by whom, and ifany  strategies were used to maintain or improve fidelity, describe them. | | | Methods: Line 286 |
| **12.ǂ** Actual: If intervention adherence or fidelity was assessed, describe the extent to whichthe  intervention was delivered as planned. | | | N/A |

** **Authors** - use N/A if an item is not applicable for the intervention being described. **Reviewers** – use ‘?’ if information about the element is not reported/not sufficiently reported.

† If the information is not provided in the primary paper, give details of where this information is available. This may include locations such as a published protocol or other published papers (provide citation details) or a website (provide the URL).

ǂ If completing the TIDieR checklist for a protocol, these items are not relevant to the protocol and cannot be described until the study is complete.

- WestronglyrecommendusingthischecklistinconjunctionwiththeTIDieRguide(see*BMJ*2014;348:g1687)whichcontainsanexplanationandelaborationforeachitem.
- ThefocusofTIDieRisonreportingdetailsoftheinterventionelements(andwhererelevant,comparisonelements)ofastudy.Otherelementsandmethodologicalfeaturesof studies are covered by other reporting statements and checklists and have not been duplicated as part of the TIDieR checklist. When a **randomised trial** is being reported, the TIDieRchecklistshouldbeusedinconjunctionwiththeCONSORTstatement(seewww.consort‐statement.org)asanextensionof**Item5oftheCONSORT2010Statement.**

When a **clinical trial protocol** is being reported, the TIDieR checklist should be used in conjunction with the SPIRIT statement as an extension of **Item 11 of the SPIRIT 2013 Statement** (see www.spirit‐statement.org). For alternate study designs, TIDieR can be used in conjunction with the appropriate checklist for that study design (see www.equator‐network.org).
