# Supplementary material for: Progressive active mobilization with dose control and training load in critically ill patients (PROMOB): Protocol for a randomized controlled trial
Source: PLoS One. 2020 Sep 3;15(9):e0238352. doi: 10.1371/journal.pone.0238352 (PMC7470388; doi:10.1371/journal.pone.0238352)
Supplement: S3 File — (DOCX) [file pone.0238352.s004.docx]

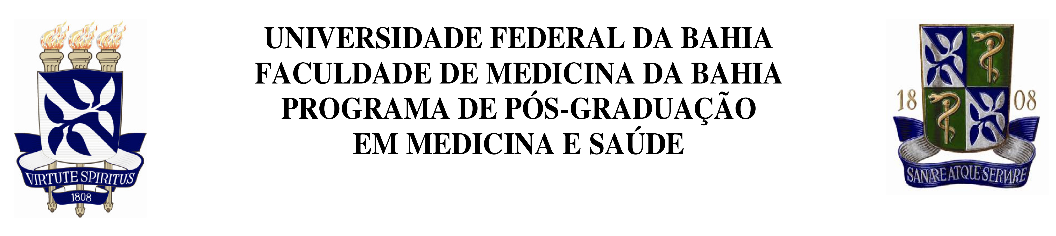


**MANSUETO GOMES NETO**

**Efeitos da adição de um protocolo de mobilização progressiva com controle de dose e carga de treinamento aos cuidados fisioterapêuticos usuais em pacientes internados em UTI: ensaio clínico randomizado**

**Salvador 2019**

**SUMÁRIO**

[1 INTRODUÇÃO 6](#_Toc535857035)

[2 JUSTIFICATIVA 9](#_Toc535857036)

[3 OBJETIVOS 10](#_Toc535857037)

[3.1 OBJETIVO GERAL 10](#_Toc535857038)

[3.2 OBJETIVOS ESPECÍFICOS 10](#_Toc535857039)

[4 MATERIAIS E MÉTODOS 11](#_Toc535857040)

[4.1 TIPO DE ESTUDO 11](#_Toc535857041)

[4.2 LOCAL 11](#_Toc535857042)

[4.3 AMOSTRA 12](#_Toc535857043)

[4.3.1 CRITÉRIOS DE ELEGIBILIDADE 12](#_Toc535857044)

[4.3.2 GRUPOS 12](#_Toc535857045)

[4.4 CÁLCULO AMOSTRAL 13](#_Toc535857046)

[4.5 PROCEDIMENTOS DE AVALIAÇÃO 13](#_Toc535857047)

[4.5.1 INDICADORES DE CUSTOS EM SAÚDE 14](#_Toc535857048)

[4.5.2 FORÇA MUSCULAR 15](#_Toc535857049)

[4.5.3 MASSA MUSCULAR 16](#_Toc535857050)

[4.5.4 FRAQUEZA MUSCULAR ADQUIRIDA NA UTI 17](#_Toc535857051)

[4.5.5 ACELEROMETRIA 17](#_Toc535857052)

[4.5.6 INDICADORES DE CAPACIDADE FUNCIONAL 19](#_Toc535857053)

[4.5.6.1 Escalas Funcionais 19](#_Toc535857054)

[5 19](#_Toc535857055)

[5.1.1.1 Timed Up and Go test 19](#_Toc535857056)

[5.1.1.2 Teste de caminhada de 6 min 19](#_Toc535857057)

[5.1.2 QUALIDADE DE VIDA 20](#_Toc535857058)

[5.1.3 ATIVIDADE E PARTICIPAÇÃO 21](#_Toc535857059)

[5.1.4 CALORIMETRIA INDIRETA 21](#_Toc535857060)

[5.1.5 TESTES DE FUNÇÃO PULMONAR 22](#_Toc535857061)

[5.2 PROCEDIMENTOS DE INTERVENÇÃO 24](#_Toc535857062)

[5.3 CRITÉRIOS DE SEGURANÇA 27](#_Toc535857063)

[5.4 DESFECHOS 28](#_Toc535857064)

[6 TRADUÇÃO, ADAPTAÇÃO TRANSCULTURAL, VALIDAÇÃO E AVALIAÇÃO DAS PROPRIEDADES PSICOMÉTRICAS 28](#_Toc535857065)

[7 VALIDAÇÃO DE DISPOSITIVO DE BAIXO CUSTO PARA AVALIAÇÃO DE ACELEROMETRIA 30](#_Toc535857066)

[8 ANÁLISE ESTATÍSTICA 30](#_Toc535857067)

[9 ASPECTOS ÉTICOS 31](#_Toc535857068)

[10 ETAPAS DA PESQUISA E CRONOGRAMA 34](#_Toc535857069)

[11 ORÇAMENTO 35](#_Toc535857070)

[12 IDENTIFICAÇÃO DA EQUIPE DE PESQUISADORES PARTICIPANTES 39](#_Toc535857071)

[13 DISPONIBILIDADE EFETIVA DE INFRA-ESTRUTURA E DE APOIO TÉCNICO PARA O DESENVOLVIMENTO DO PROJETO 41](#_Toc535857072)

[14 REFERÊNCIAS 42](#_Toc535857073)

[15 APÊNDICE 48](#_Toc535857074)

**Índice de Quadros**

[**Quadro 1** - Resumo dos momentos e variáveis de avaliação desde a internação na UTI até 12 meses da alta hospitalar](#_41mghml) 24

[**Quadro 2 -** Protocolo de mobilização progressiva estratificado por níveis funcionais e desempenho.](#_2grqrue) 26

[**Quadro 3** - Critérios para interrupção da mobilização precoce de pacientes internados na UTI. Fonte: Adler e Malone (2012) ^11^.](#_vx1227) 27

# INTRODUÇÃO

Nos últimos anos houve grandes avanços científicos e tecnológicos nos cuidados aos pacientes internados em Unidade de Terapia Intensiva (UTI), o que acarretou numa importante redução das taxas de mortalidade ^1^. No entanto, os pacientes sobreviventes podem desenvolver grave incapacidade funcional que pode perdurar até 5 anos após a alta hospitalar ^2^.

Essa incapacidade funcional é um resultado da soma de fatores agravantes como sepse, sedação, bloqueio neuromuscular, inflamação, hiperglicemia, com destaque para a imobilidade prolongada no leito ^3,4^. A combinação de imobilidade e catabolismo, frequentemente observada nesses pacientes, pode levar disfunção neuromuscular significativa e prologada com incidência de até 25% ^2^, impactando em baixa qualidade de vida e aumento dos custos em saúde ^5,6^.

A implementação de estratégias de combate a imobilidade é denominada mobilização precoce (MP). A MP em UTI é um termo amplo que engloba exercícios ativos de membros, transferências no leito realizadas ativamente, sedestação à beira do leito, transferência para cadeira, ortostatismo e deambulação ^7^, onde o paciente utiliza sua própria força e controle muscular, ainda que necessite de assistência da equipe multidisciplinar ou de equipamentos ^6,8^.

Dentro desse contexto, houve crescente interesse na avaliação da viabilidade, segurança e efeitos fisiológicos associados a MP de pacientes internados em UTI ^9,10^. Diversos estudos investigaram a eficácia da MP, resultando em revisões sistemáticas recentes que reforçam a segurança e a importância da implementação dessa prática, contribuindo com melhora da capacidade funcional e possibilidade de redução do tempo de internação hospitalar e aumento de sobrevida ^6,8,11^.

Estudos que investigam a eficácia da MP em UTI começaram a surgir na literatura nos últimos 20 anos ^8^, isso, pois no passado, os pacientes eram sedados profundamente e submetidos a longo períodos de imobilidade no leito. O repouso no leito era utilizado como estratégia terapêutica, pois o entendimento era de que o exercício poderia causar eventos adversos, apesar das evidências de que essa prática resultava em descondicionamento físico importante após a alta ^6,12^.

Atualmente existe o consenso, reportado através de revisões sistemáticas e metanálises ^6,8,11,13^, que são mínimos os efeitos adversos associados a MP (≤4%) ^11^, além disso, a maioria destes eventos adversos são transitórios e benignos ^6^. Assim, surgiram os primeiros critérios objetivos para a segurança da mobilização de pacientes adultos em UTI, que envolveram principalmente variáveis hemodinâmicos e respiratórias ^9^, e foram posteriormente endossados pela European Respiratory Society and European Society of Intensive Care Medicine ^14^. Em 2012 foi publicada uma revisão sistemática sobre o tema, sendo incluídos parâmetros de segurança neurológicos e apresentados os principais pontos de corte para variáveis hemodinâmicas e respiratórias ^11^.

Em 2014 uma nova revisão sistemática, seguida por uma reunião multidisciplinar de 23 especialistas em UTI, apresentou um consenso de critérios de segurança para MP (Expert consensus and recommendations on safety criteria for active mobilization of mechanically ventilated critically ill adults) ^6^. O consenso foi avaliado e aprovado numa reunião internacional envolvendo 94 especialistas multidisciplinares em UTI. Os critérios de segurança foram resumidos em quatro categorias (respiratória, cardiovascular, neurológica e outras) através de um sistema de padrão de luzes de trânsito: vermelho indicaria a necessidade de cautela, com o risco de um evento adverso; amarelo indicaria que a mobilização era possível, mas somente após uma análise mais aprofundada entre a equipe multidisciplinar da UTI; e verde indicaria que o paciente estava seguro para ser mobilizado ^6^. Foi acordado que a presença de um sinal vermelho deveria seria suficiente para alertar sobre o potencial risco de um evento adverso durante a mobilização, mesmo se todos os outros parâmetros fossem verdes. Assim, ao considerar a decisão de mobilizar um paciente, os critérios devem ser avaliados no momento da mobilização, mas mudanças nas condições e na direção das tendências das variáveis nas horas precedentes também devem ser consideradas ^6^.

Em paralelo aos avanços dos critérios de segurança, novos estudos procuraram identificar quais eram os métodos de prescrição de exercícios por fisioterapeutas em UTIs. Skinner *et al,* ainda no ano de 2008, já destacava que existia pouca fundamentação e alertava sobre a necessidade de mais pesquisas para estabelecimento de uma dose e carga de treinamento ideal ^5^. Mesmo após a realização de vários ensaios clínicos sobre a MP, o tema dose de mobilização ainda hoje não está claro. A carga de treinamento tanto em termos de volume e intensidade não são bem monitoradas. Uma prova substancial disso é que na conclusão de um importante estudo de atualização de revisão sistemática em 2013, os autores deixam claro a necessidade de determinar a dosagem ótima de intensidade, duração e frequência da mobilização em UTI ^8^.

Mesmo se passado 4 anos dessa publicação, ainda não são encontrados protocolos de mobilização com descrição estruturada de dose e controle de carga de treinamento. Poucos ensaios clínicos apresentaram dados sobre frequência e duração da MP, e nenhum dos estudos incluídos numa revisão mais recente relatou informações adequadas sobre a intensidade dos exercícios, sendo que apenas estudos que utilizam-se de tecnologias como o cicloergômetro relatam cargas de treinamento com maior integralidade de descrição ^13^.

Num estudo pioneiro na avaliação da eficácia da MP, apesar de não discutir mais profundamente sobre aspectos relativos a controle de dose e de carga de treinamento, pôde-se observar que os pacientes do grupo protocolo foram mobilizados com maior frequência (80% vs. 47%, p < 0,001) e iniciaram a mobilização mais frequentemente ainda na UTI (91% vs. 13%, p < 0,001), quando comparado com o grupo de cuidados habituais ^15^. A partir de então, pôde-se observar que houve uma tendência no aumento da frequência diária da MP, de uma vez ao dia ^15^ para duas vezes ao dia ^16^, sendo que parâmetros como duração e intensidade são pouco debatidos e não são monitorados objetivamente.

Um estudo observacional recente, com um protocolo mais robusto, envolvendo dispositivos tecnológicos, maior duração e frequência diária da mobilização, mostrou-se seguro, e os pacientes relataram experiências muito positivas e sentimentos de bem-estar durante várias modalidades de sessões de fisioterapia ^16^. Como alternativa para tentar examinar e discutir melhor a dose de tratamento os autores incorporaram a escala de percepção subjetiva de esforço de Borg ^17^, escores de satisfação com a mobilização e sensação subjetiva de dor ^16^. Entretanto ainda não existe um ensaio clínico randomizado com metodologia adequada ^18^ que avalie o impacto dessas simples estratégias de estruturação de controle de dose e carga de treinamento, principalmente com dispositivos de mensuração objetiva do nível de atividade física como acelerômetros ^19^.

# JUSTIFICATIVA

Apesar dos conhecimentos de segurança e dos benefícios da mobilização precoce (MP) em UTI, existem lacunas dentro do que tange a dose de tratamento ótima. Talvez a dificuldade em desenhar estudos com controle adequado de dose e carga de treinamento resida em especificidades próprias dentro do contexto de terapia intensiva, onde só mais recentemente houve o entendimento dos graves malefícios da cultura do repouso no leito como estratégia terapêutica. Entretanto, com o conhecimento atual de parâmetros de segurança e com diversos protocolos de exercícios já publicados, existe a necessidade de uma monitorização maior das variáveis de tipo, intensidade, frequência e duração da MP.

Princípios metodológicos simples, como adição de escalas de sensação subjetiva de esforço, dor e satisfação, associadas a padrões bem documentados de frequência, tipo, intensidade e duração, já amplamente utilizados em nível ambulatorial através da acelerometria, podem tornar a prescrição de exercícios em UTI mais sistematizada. Assim, os resultados deste estudo podem contribuir para prevenção da Fraqueza Muscular Adquirida na UTI e da incapacidade funcional após a alta hospitalar, reduzindo custos em saúde e melhorando a qualidade de vida de pacientes sobreviventes a doença crítica.

# OBJETIVOS

## OBJETIVO GERAL

- Identificar se a adição de um protocolo de mobilização progressiva com controle de dose e carga de treinamento aos cuidados fisioterapêuticos usuais é eficaz na redução do tempo de internação e na melhora da funcionalidade em pacientes internados em UTI

## OBJETIVOS ESPECÍFICOS

- Realizar a tradução, adaptação transcultural, validação para população brasileira e avaliação das propriedades psicométricas da escala Surgical Intensive Care Unit Optimal Mobilization Score (SOMS) e do Expert consensus and recommendations on safety criteria for active mobilization of mechanically ventilated critically ill adults

- Comparar o gasto energético do grupo intervenção (adição de um protocolo de mobilização com controle de dose e carga de treinamento) com o grupo controle (cuidados habituais de mobilização).

- Validar um dispositivo de baixo custo de acelerometria para avaliação de pacientes durante todo processo de hospitalização

- Determinar a frequência de FMA-UTI e avaliar a sua associação com indicadores de custos em saúde, força e massa muscular, acelerometria, indicadores de capacidade funcional, qualidade de vida, atividade/participação e função pulmonar

- Determinar a associação entre variáveis de funcionalidade e indicadores de custos em saúde

- Identificar os fatores associados com mortalidade e sobrevida até 12 meses da alta hospitalar

# MATERIAIS E MÉTODOS

O plano de pesquisa será elaborado em concordância com as normas vigentes para a pesquisa envolvendo seres humanos, conforme a Resolução 466/12 do Conselho Nacional de Saúde do Ministério da Saúde (CNS/MS) ^20^.

Serão adotados critérios rigorosos de biossegurança para controle de infecção conforme a Agência Nacional de Vigilância Sanitária (ANVISA) ^21^, respeitando todas as recomendações da comissão de controle de infecção hospitalar. Todos os procedimentos de avaliação e intervenção obedecerão diretrizes específicas de segurança de mobilização de pacientes críticos do Expert consensus and recommendations on safety criteria for active mobilization of mechanically ventilated critically ill adults ^6^ acrescidos de critérios objetivos para interrupção da mobilização de pacientes criticamente enfermos dispostos na revisão sistemática publicada na *Cardiopulmonary Physical Therapy Journal* ^11^, conforme descrito no tópico 4.4 (critérios de segurança).

## TIPO DE ESTUDO

Estudo unicêntrico, com randomização simples, duplo-cego, controlado por cuidados habituais de mobilização, realizado na cidade de Salvador, Bahia, Brasil. O estudo ocorrerá de acordo com as recomendações do CONSORT (Consolidated Standards of Reporting Trials) ^18^.

## LOCAL

O estudo será realizado no Hospital Universitário Professor Edgard Santos (HUPES), localizado na Rua Doutor Augusto Viana, s/n, Bairro Canela – Salvador/Ba. As avaliações dos pacientes após a alta hospitalar serão realizadas no dia do retorno para consultas médicas no Ambulatório Professor Francisco de Magalhães Neto (AMN) do HUPES, localizado na Rua Padre Feijó, nº 240, Bairro Canela – Salvador/Ba.

## AMOSTRA

Pacientes internados em unidade de terapia intensiva (UTI). Os pesquisadores identificarão os pacientes elegíveis e se aproximaram dos representantes legalmente autorizados ou dos pacientes, informarão sobre os objetivos e procedimentos, convidando-os a participar do estudo e assinarem o termo de consentimento livre e esclarecido (TCLE) (ANEXO).

### CRITÉRIOS DE ELEGIBILIDADE

Critérios de inclusão: pacientes com idade igual ou superior a 18 anos, classificação funcional mínima de nível 1, ou seja, ser capaz de rolar no leito e fazer ponte (Quadro 2), possuírem índice de Barthel ^22^ de pelo menos 70 duas semanas antes da admissão na UTI ^23^ e capacidade de interação com o fisioterapeuta, determinada por pelo menos 3 respostas positivas de 5 comandos: (1) “Abra (feche) os olhos”, (2) “Olhe para mim, (3)“ Abra a boca e ponha a língua para fora ”, (4)“ Acene com a cabeça ”, e (5)“ Levante a sua sobrancelhas quando eu tenho contado até 5 "^4^.

Critérios de exclusão: mortalidade estimada superior a 50% de acordo com Acute Physiology and Chronic Health disease Classification System II (APACHEII) ^23^, apresentem aumento de pressão intracraniana, pacientes que sofreram parada cardiorrespiratória, possuem fraturas instáveis ​​que dificulte a progressão nos níveis de mobilização, amputados de membros inferiores, com doença degenerativa neuromuscular, acidente vascular encefálico, traumatismo crânio encefálico grave, ou que realizaram radioterapia e/ou quimioterapia nos últimos 6 meses.

### GRUPOS

Após o início da pesquisa todos os pacientes admitidos na UTI serão monitorados para avaliação quanto aos critérios de elegibilidade através de visitas diárias dos pesquisadores em comunicação direta com a equipe multidisciplinar. A medida que os responsáveis assinarem o TCLE e forem verificados os critérios de elegibilidade (inclusão e exclusão), os pacientes serão alocados de forma sequencial e aleatória nos grupos intervenção e controle utilizando a estratégia de randomização simples ^24–26^. Esta técnica mantém aleatoriedade completa da atribuição de um sujeito a um grupo particular ^18,27^. Os números, seguindo a ordem gerada, serão lacrados num envelope opaco, codificado e sem identificação^28^ que serão assinados e datados pelo pesquisador responsável pela alocação.

Apenas o fisioterapeuta que realizará a intervenção possuirá conhecimento do significado do código contido no envelope, e, portanto, saberá quais pacientes receberão o protocolo de mobilização. A randomização será realizada por um pesquisador independente não envolvido no estudo (não fará parte nem da intervenção, nem da avaliação e será cego para os códigos de intervenção e controle).

Os pacientes não serão informados que estão utilizando estratégia de tratamento diferentes, serão abordados como se estivem recebendo cuidados de rotina em UTI. Os avaliadores também não terão conhecimento da alocação dos pacientes. A tabulação e tratamento dos dados também será cega. Essa estratégia utilizada caracterizará o estudo como duplo cego.

## CÁLCULO AMOSTRAL

Uma amostra de 104 pacientes, 52 por grupo, foi calculada considerando um poder de 80% e nível de significância 5%, com uma diferença média clinicamente significativa de 3,7±2,6 dias ^29^ para o tempo de internação na UTI entre os grupos intervenção e controle.

## PROCEDIMENTOS DE AVALIAÇÃO

Serão coletados dados sóciodemográficos, de condição de saúde e estilo de vida prévios dos pacientes através do relato dos responsáveis legais, e quando possível, dos próprios pacientes. Os aspectos clínicos de interesse do estudo serão coletados através do prontuário do paciente, monitores da UTI e comunicação direta com a equipe multidisciplinar. Os dados serão registrados num formulário próprio (APÊNDICE) por pesquisadores devidamente treinados.

O exame do paciente à beira do leito e realização de testes funcionais será realizado por pesquisadores cegos em relação aos grupos protocolo e intervenção. Não poderá haver avaliação e coleta de dados clínicos nos horários em que o fisioterapeuta que implementa a intervenção estiver presente no serviço.

Os dados coletados serão relativos aos aspectos sociodemográficos, indicadores de custos em saúde, diagnóstico, aspectos farmacológicos, exames complementares, nutrição, sinais vitais, sinais de infecção/inflamação, parâmetros ventilatórios, uso de traqueostomia, ventilação não invasiva, sucesso/falha de desmame e extubação e fatores limitantes da mobilização. Serão realizadas medidas antropométricas, aplicação do score de gravidade APACHE II, medida de força muscular manual e dinamômetros, medida massa muscular através do ultrassom, medida de gasto energético através de calorimetria indireta, medida da acelerometria através de sensores fixados a coxa do paciente e de análise de vídeo, realização de testes funcionais (Timed Up and Go test, Teste de caminhada de 6 min), avaliação da função pulmonar (espirometria e manovacuometria), escores de funcionalidade, qualidade de vida e atividade e participação.

### INDICADORES DE CUSTOS EM SAÚDE

Tempo de internação na UTI: Será registrado em horas contadas a partir da admissão até o horário da saída do paciente da UTI.

Tempo de internação hospitalar: Será registrado em horas contadas a partir da admissão até o horário de saída do paciente do hospital.

Tempo de ventilação mecânica: Será registrado em horas contadas a partir da intubação até a extubação.

Infecção Hospitalar: infecção adquirida após a admissão do paciente na unidade hospitalar e que se manifesta durante a internação ou após a alta, quando puder ser relacionada com a internação ou procedimentos hospitalares ^21^, constante no prontuário dos pacientes.

Dias livres: Serão contabilizados o número de dias livres do hospital, UTI, suporte ventilatório, suporte cardiovascular e renal de acordo com as recomendações dos *End points for phase II trials in intensive care* ^30^.

### FORÇA MUSCULAR

Para avaliação da força muscular os pacientes deverão estar alertas, ou seja, com capacidade de responder positivamente a 3 de 5 comandos: (1) “Abra (feche) os olhos”, (2) “Olhe para mim, (3)“ Abra a boca e ponha a língua para fora ”, (4)“ Acene com a cabeça ”, e (5)“ Levante a sua sobrancelhas quando eu tenho contado até 5 "^4^.

. Esse procedimento visa garantir que existe grau de cooperação suficiente dos pacientes para realização dos movimentos necessários ^4^. Antes do início do estudo, os dinamômetros serão calibrados pelo fabricante.

A força muscular será avaliada por um único pesquisador, de 3 em 3 dias ^31^ até a alta hospitalar, a contar do primeiro dia em que o paciente apresentou-se alerta.

- Medical Research Council sum-score (MRC): Serão avaliados seis grupos musculares bilaterais através de movimentos específicos (abdução de membro superior, flexão de cotovelo, extensão de punho, flexão de quadril, extensão de joelho. A força muscular é pontuada de 0 a 5 (0= sem contração visível ou palpável; 1= contração visível ou palpável sem movimento do membro; 2= movimento do membro, mas não contra a gravidade; 3= movimento contra a gravidade (próximo da amplitude de movimento passiva), mas não contra resistência; 4= movimento contra a gravidade e resistência (arbitrariamente julgada para ser submáximo para o sexo e idade); 5= movimento normal. Um somatório final igual a 60 identifica força muscular normal, sendo que 0 caracteriza tetraplegia ^4,32,33^. Os testes serão realizados numa ordem fixa e cada movimento será realizado passivamente pela primeira vez. Inicialmente o paciente estará posicionado em supino à 45º, seguindo o protocolo (<http://links.lww.com/CCM/A781>) apontado na última revisão sistemática sobre o tema ^33^.

**-** Força de Preensão Manual (FPM): Será avaliada apenas se o paciente obtiver um escore mínimo de 3, bilateralmente, no MRC (movimentos de extensão de punho e flexão de cotovelo) ^32^. Para medida da FPM será utilizado um dinamômetro hidráulico (Saehan Corporation SH5001, Korea). O indivíduo, após escolher o braço considerado mais forte, será posicionado adequadamente, o mais próximo de em 90º, com ombro aduzido, cotovelo fletido a 90° ^34^, antebraço em posição neutra, e o punho entre 0 a 30^o^ de extensão. Os pacientes, através de um forte comando verbal do examinador, serão estimulados a desenvolver força máxima, pressionando a alça do dinamômetro, que pode ser apoiado pelo examinador, por pelo menos 6 segundos (s), em 3 tentativas, com intervalo de 1 minuto. O maior valor de força de preensão manual em quilograma força (Kgf) será considerado para a análise ^32–34^.

- Handheld dynamometria (HD): Será realizado conjuntamente à avaliação do MRC, obedecendo os mesmos critérios de posicionamento, através de um dinamômetro manual (modelo 01163; Lafayette Instrument, Ind). Serão avaliados a flexão de cotovelo e extensão de joelho. O examinador deve manter o dinamômetro parado enquanto o paciente exerce força máxima contra ele ^35^. Os movimentos serão realizados 3 vezes, com intervalo de 1 minuto entre as medidas. Uma placa rígida será colocada sob o membro a ser testado e o dinamômetro posicionado de forma perpendicular ^36^.

- Teste de sentar e levantar de uma cadeira 5 vezes: Trata-se de um teste que possui correlação positiva com a força de membros inferiores. Inicialmente os indivíduos serão avaliados quanto a capacidade de levantar de uma cadeira com encosto reto e colocada ao lado de uma parede. Os participantes deverão estar com os braços cruzados. Se for bem sucedido, os participantes serão convidados a se levantar e sentar cinco vezes, o mais rápido possível, e o tempo será cronometrado a partir da posição inicial sentado até a posição final em pé no final da quinta execução ^37^.

### MASSA MUSCULAR

O ultrassom muscular permite a detecção de atrofia bem como mudanças arquitetônicas e fasciculação ^38^. O indicador de massa muscular será obtido apela área seccao transversal do reto femoral medida através de ultrassonografia ^39^ em modo B, utilizando um sistema de matriz linear de 8 MHz de 5,6 cm (PLM805, Toshiba Medical Systems, Crawley, UK). Será utilizada uma técnica planimétrica após a linha ecogênica interna do reto femoral ser delineada por um cursor móvel sobre uma imagem congelada (3 medidas consecutivas, desde que não haja mais que 10% de variação entre as medidas).

O transdutor será colocado perpendicular ao longo eixo da coxa no ponto mais alto em que toda a secção transversal do reto femoral possa ser visualizada em um único campo (três quintos da distância da parte anterior da espinha ilíaca superior à borda patelar superior). O paciente estará em decúbito dorsal com a perna apoiada em extensão passiva. A avaliação da secção transversa do bíceps braquial será realizada no membro dominante, em posição anatômica, sendo a secção transversa medida no ponto da distância média da tuberosidade maior da cabeça do úmero (acrômio) até o olecrano (fossa olecranóide). A frequência será de 7,5 Mhz, no modo B, com o transdutor linear e perpendicular a estrutura.

O gel de contato será aplicado para minimizar a distorção subjacente dos tecidos moles. A Imagem oblíqua será minimizada pelo operador usando feedback visual para obter a menor imagem em secção transversal. A profundidade de digitalização será ajustada de acordo com o discernimento de orientação femoral. Manobras de contração-relaxamento poderão ser utilizada para delinear os septos musculares antes da aquisição da imagem ^40^.

A massa muscular será avaliada por um único pesquisador nas primeiras 24 horas de inclusão dos pacientes no estudo, na alta da UTI e imediatamente antes da alta do hospital.

### FRAQUEZA MUSCULAR ADQUIRIDA NA UTI

A fraqueza muscular adquirida na UTI (FAM-UTI) é um efeito prejudicial da doença crítica na função física. Este termo refere-se a uma grande variedade de distúrbios caracterizados por início agudo de comprometimento neuromuscular para o qual não há outra causa plausível do que a doença crítica. É caracterizada por fraqueza e flacidez simétrica dos membros, que é mais acentuada nos músculos proximais do que nos músculos distais. Os músculos faciais e oculares são frequentemente poupados. As diretrizes atuais recomendam o diagnóstico através da avaliação clínica da força muscular realizada pelo somatório da pontuação do Medical Research Council sum-score (MRC). Uma pontuação de MRC abaixo de 48 (pontuação máxima de 60) confere o diagnóstico de FAM-UTI ^41^.

### ACELEROMETRIA

O monitoramento preciso da atividade física pode ser obtido através de acelerômetro. Em pacientes críticos, hospitalizados e com pouca mobilidade essa monitorização pode contribuir para um adequada controle da carga de treinamento ^19^. Através dos dados exportados para uma planilha eletrônica, serão calculados o gasto energético ^42^, número diário de passos, tempo de atividade e repouso, tempo gasto em atividade física, equivalentes metabólicos médios do nível de tarefa ^43^. Serão avaliados a quantidade de atividade estática / dinâmica, quantidade de tempo gasto na posição posição deitada, sentada, número de passos e número de transições diárias ^44^.

Para avaliação será utilizado o *ActivPALTM Professional* que foi adotado em trabalho semelhante anteriormente ^44^. O dispositivo será utilizado 24 h por dia durante todo período de internação, exceto no final de semana. Antes do início do estudo, o sensor será programado com a introdução de dados do paciente (sexo, idade e altura), para capturar o tempo de atividade física (definido como tempo acumulado de permanência > 1 MET – taxa metabólica de repouso), e o gasto energético para dias e horários individuais ^42,43^. Para o grupo intervenção ainda serão calculados os picos de aceleração, aceleração média, cadência, potência e declínio de potência durante todos os exercícios do protocolo.

O activPALTM será fixado à coxa do paciente na metade da distância entre o quadril e o joelho na orientação correta conforme delineado pelo fabricante. Será utilizado um adesivo PALstickiesTM e cobertura com um curativo adicional recomendado para suporte extra (fita cirúrgica Medipore 3M). Este local de fixação será o escolhido, pois é proximal ao centro de massa do corpo e, portanto, melhor representativo do movimento total do corpo. Os dados brutos do acelerômetro serão salvos num cartão de memória removível interno do registrador de dados. O activPALTM será programado através de sua interface de computador e seu relógio interno sincronizado com o do dispositivo.

Diariamente serão monitorados a integridade da pele e o correto posicionamento do sensor. A análise de dados ocorrerá de acordo com as diretrizes recomendadas para a elaboração de relatórios objetivos de atividade física ^42^. Após a conclusão da gravação, os dados do activPALTM serão descarregados através da sua interface de computador e exportados para uma planilha do Excel.

### INDICADORES DE CAPACIDADE FUNCIONAL

Existem diversos indicadores de capacidade funcional, desde testes de desempenho específicos até escalas pontuadas através de observação direta das atividades.

#### Escalas Funcionais

Para a estratificação em níveis funcionais será utilizado Surgical Intensive Care Unit Optimal Mobilisation Score ^23,45^; para avaliação da mobilidade será utilizado o Functional Status Score for the Intensive Care Unit ^46^; o status funcional prévio será avaliado através do índice de Barthel ^22^. Os momentos de avaliação estão especificados no quadro 1.

####

#### Timed Up and Go test

Para a realização do teste "Time Up and Go" (TUG) será necessário uma cadeira, um cronômetro, fita métrica e uma ficha para anotações dos dados. O teste será realizado solicitando o paciente a levantar de uma cadeira, andar por 3 metros girar e retornar a cadeira e por fim sentar, será mensurado o tempo gasto (em segundos) para completar a atividade.. Os momentos de avaliação estão especificados no quadro 1.

#### Teste de caminhada de 6 min

O teste de caminhada de seis minutos (TC6) mede a distância percorrida enquanto o indivíduo é instruído a caminhar o mais rápido que consiga em seis minutos. Ele avalia as respostas global e integrada dos sistemas envolvidos durante o exercício, incluindo os sistemas cardiovascular, respiratório e neuromuscular unidades neuromusculares e o metabolismo muscular. Para a maioria dos indivíduos, o TC6 é um teste submáximo da capacidade funcional, pois a pessoa escolhe sua própria intensidade de exercício, sendo permitido que pare e descanse durante sua execução. Grande parte de nossas atividades da vida diária é realizada em níveis submáximos, sendo assim, o TC6 reflete bem a capacidade funcional para as atividades de vida diária ^48^.

Para a aplicação do TC6, será utilizado o protocolo da ATS Statement: Guidelines for the Six-Minute Walk Test ^48^. O TC6 será realizado em uma pista plana e rígida de no mínimo 30 metros de comprimento e um metro e meio de largura, com marcações a cada metro de distância com piso antiderrapante, em local sem passagem de outras pessoas, silencioso, bem iluminado e arejado. Antes de realizar o teste da caminhada, o paciente será instruído sobre como ele será realizado e que, caso apresente desconforto respiratório muito grande, dor no peito ou dor muscular intensa, pode diminuir a velocidade e até mesmo parar. Caso isso aconteça, o cronômetro permanecerá acionado. O paciente será instruído a andar de forma habitual sem incentivo do examinador. Durante a realização do teste, no 2º, 4º e 6º minuto o examinador caminhará discretamente atrás do paciente, e não ao lado de cada participante, para evitar influenciar a velocidade de marcha selecionada pelo paciente, mostrando-lhe escala previamente conhecida por eles, onde teriam que escolher uma opção de percepção do esforço presente na tabela, conhecida com escala de Borg. Ao final de seis minutos, a distância percorrida será registrada. Ao início e ao término do teste serão monitorizadas a pressão arterial. A freqüência cardíaca e respiratória serão monitoradas durante toda a realização do teste ^48^.

O cálculo do valor previsto ou de referência para distância no TC6min é realizado como proposto por, *Enright e Sherril* ^49^*:* em homens (distância): (7,57 x altura cm) – (5,02 x idade) – (1,76 x peso Kg) – 309e em mulheres (distância):(2,11 x altura cm) – (2,29 x peso Kg) – (5,78 x idade) + 667m. Os momentos de avaliação estão especificados no quadro 1.

### QUALIDADE DE VIDA

Os indicadores de QV serão medidos por meio da aplicação do “*Short-Form 36 - Medical Outcomes* *Study*” (SF36), já adaptado e validado no Brasil^.^ O SF36 é um questionário multidimensional formado por 36 itens, englobados em oito escalas ou componentes: capacidade funcional, aspectos físicos, dor, estado geral de saúde, vitalidade, aspectos sociais, aspectos emocionais e saúde mental. Apresenta um escore final de zero a 100, no qual zero corresponde ao pior estado e 100 ao melhor estado ^50^. Os momentos de avaliação estão especificados no quadro 1.

### ATIVIDADE E PARTICIPAÇÃO

A World Health Organization Disabilty Assessment Schedule 2.0 (WHODAS 2.0) é um instrumento desenvolvido pela Organização Mundial de Saúde (OMS) para avaliar as limitações nas atividades e restrições da participação já adaptado e validado no Brasil ^51^. No WHODAS 2.0 a incapacidade é entendida independentemente de um quadro de doença ou condição de saúde prévia. O WHODAS 2.0 foi desenhado para avaliar a funcionalidade em 6 domínios de atividade: Cognição, Mobilidade, Auto-cuidado, Relações Interpessoais, Atividades Diárias e Participação. Em cada domínio, as questões serão respondidas considerando os últimos 30 dias da vida diária do indivíduo. Os participantes serão ainda questionados quanto ao nível de dificuldade experimentado, tendo em conta a forma como executa a atividade, incluindo o uso de um qualquer produto de apoio. Os resultados são normalizados podendo variar entre o valor zero e o valor cem. Os momentos de avaliação estão especificados no quadro 1.

### CALORIMETRIA INDIRETA

Trata-se de um método não invasivo para análise de consumo de oxigênio e, portanto, estimativa do gasto energético. As medidas serão obtidas em todos os pacientes com o calorímetro portátil (Deltatrac II^®^ Metabolic Monitor - Datex-Ohmeda, Finlândia). Será realizada uma calibração inicial, antes do protocolo de estudo, com o teste de queima do álcool, de acordo com especificações do fabricante. Antes de cada estudo de troca de gases, o calorímetro será aquecido por 30min e a seguir calibrado com uma mistura conhecida de gases contendo 5% de dióxido de carbono (CO2 ) e 95% de O2 . A linha de coleta de gases inspiratórios será posicionada na saída do ramo inspiratório do ventilador mecânico. Todo o ar expirado será coletado da via expiratória do ventilador. Os indivíduos em ventilação espontânea serão avaliados através de um bocal. As medidas serão realizadas 30 minutos antes e 30 após as intervenções de mobilização, sejam elas de cuidados habituais ou do protocolo de intervenção para efeito de comparação de gasto energético durante o estudo piloto.

Todos estes procedimentos serão realizados, de acordo com as especificações fornecidas pelo fabricante. Com o paciente estável, sem manipulação das vias aéreas nem dos parâmetros do respirador, durante pelo menos 30min, serão feitas 30 medições minuto a minuto do VO2 (mL.min-1). Ao final de cada período de 30 min será calculado a média e o desvio padrão do consumo de oxigênio ^52,53^.

### TESTES DE FUNÇÃO PULMONAR

- Força Muscular Respiratória: Será inferida através das medidas das pressões respiratórias estáticas máximas (PRMs), por meio do Manovacuômetro Digital MVD 300. Será utilizada como interface aparelho-paciente uma peça bucal descartável e um filtro barreira isolador de pressão de uso individual. As mensurações das PRMs conduzidas conforme diretrizes específicas ^54^. Os valores das medidas serão armazenados no software MVD 300 system. As manobras serão válidas quando tecnicamente aceitáveis (sem vazamentos e com duração de pelo menos 2 segundos) e reprodutíveis (com valores que não difiram entre si mais de 10% do valor mais elevado). Os esforços devem ser mantidos por pelo menos 2s para que a pressão sustentada máxima seja registrada após o primeiro segundo. O período de recuperação entre as manobras será de 1min e todos os pacientes realizarão até cinco manobras, podendo extrapolar caso o valor mais alto surja na última tentativa. Os resultados dos exames serão obtidos quando houver três esforços aceitáveis com pelo menos dois reprodutíveis entre eles, selecionando assim o maior valor inspiratório e expiratório para a análise.

As manobras serão divididas em: (1) *Pressão Inspiratória Máxima (PImáx)* - será orientado que o paciente respire tranquilamente com o bucal acoplado na boca até que seja solicitado uma exalação lenta e máxima a nível do volume residual (VR). A partir de então o orifício de oclusão será bloqueado pelo examinador e solicitado um esforço inspiratório máximo até que seja autorizado o término da manobra. A instrução verbal é: “Jogue todo o ar de dentro do peito para fora e puxe novamente para dentro com toda sua força”. Será registrado a maior pressão (cmH2O) gerada após o primeiro segundo do início das manobras; (2) *Pressão Expiratória Máxima (PEmáx) -* Seguiram-se os mesmos procedimentos da PImáx para a PEmáx, porém, com a solicitação de uma inspiração máxima até a capacidade pulmonar total (CPT). Quando o orifício de oclusão for fechado, o examinador deverá segurar as bochechas dos pacientes para eliminar a ação dos músculos bucinadores. O comando será: “Encha bem o peito de ar até seu máximo e sopre com toda sua força”. Será registrado a maior pressão (cmH2O) gerada após o primeiro segundo do início das manobras. Além do pico de pressão, serão calculadas o tempo da rampa de subida da PRMs.

- Espirometria: A verificação dos parâmetros espirométricos será realizada através do MicroLab™ Spirometer (Care Fusion - USA), devidamente calibrado, respeitando as Diretrizes da Sociedade Brasileira de Pneumologia e Tisiologia ^55^. O MicroLab™ Spirometer é um espirômetro de fluxo que preenche as condições exigidas para precisão e acurácia da American Thoracic Society (ATS) e da European Respiratory Society (ERS) ^56^. A espirometria será realizada na posição sentada, com coluna ereta, pés apoiados no chão, sem apoio para os membros superiores, usando um clipe nasal e repousando entre cinco a dez minutos antes do teste. Haverá intervalo de um minuto entre as medidas (MILLER et al., 2005). No mínimo três e no máximo oito manobras expiratórias forçadas serão realizadas, sendo os testes realizados por um único pesquisador. O paciente será orientado a inspirar profundamente até CPT e expirar tão rápida e intensamente quanto possível até o VR. O pesquisador, com auxílio do bocal, demonstrará entusiasticamente todos os passos da manobra e fornecerá o comando: “O Sr. (a) vai puxar o máximo de ar até encher o peito completamente e depois vai colocar este aparelho (bocal acoplado ao transdutor) na boca, fechar os lábios completamente e soprar bem forte, e vai continuar soprando até que eu mande parar e puxar o ar fundo novamente”. Telas de incentivo fornecidas pelo espirômetro serão utilizadas. Sucessivas demonstrações das manobras serão realizadas pelo pesquisador. A CVF (L) e o VEF1 (L) serão selecionados de acordo com os critérios para aceitação e reprodutibilidade das curvas espirométricas ^56^. Para aceitação inicial de um teste espirométrico, deve-se proceder com inspeção visual do indivíduo e das curvas fluxo/volume e fluxo/tempo geradas na tela do espirômetro. O início da expiração deve ser abrupto e sem hesitação, com evidência de esforço máximo, término e duração satisfatória (tempo expiratório, em geral, maior que 6s ou platô no último segundo), ausência de artefatos como tosse, vazamento perioral, obstrução da peça bocal, manobra de valsalva e/ou ruído glótico.

Os momentos de avaliação da função pulmonar estão especificados no quadro 1.

| **Avaliação de linha de base**   - Diagnósticos, dados demográficos e antropométricos; Apache II e mortalidade estimada ^23^; tempo de internação e de suporte ventilatório antes da admissão na UTI - Classificação funcional (Quadro 2); Índice de Barthel recordatório (avaliado através do representante legal) duas semanas antes da admissão na UTI ^22^; capacidade de interação ^4^; - Força e massa muscular; acelerometria; Score SOMS e FSS |
| --- |
| **Avaliação na UTI e durante a internação hospitalar**   - Sensação de esforço ^17^; Satisfação com a atividade ^16,42^ - Critérios de segurança ^6,11^; Fatores limitantes da mobilização; Nível funcional e desempenho (Quadro 2) - Calorimetria (apenas na UTI) - Parâmetros ventilatórios; dose diária e número de dias recebendo medicações; sucesso no desmame; sucesso na extubação; tempo de início da mobilização; número de sessões de mobilização; tempo do primeiro dia de saída do leito, aspectos nutricionais, sinais de infecção e inflamação |
| **Na alta da UTI**   - Tempo de prontidão para alta da UTI; Tempo de internação na UTI - Força muscular e massa muscular; Indicadores de capacidade funcional |
| **Imediatamente antes da alta hospitalar**   - Tempo de internação hospitalar; tempo de ventilação mecânica; infecção hospitalar; função pulmonar; força e massa muscular; Indicadores de capacidade funcional |
| **Após a alta hospitalar**   - Dias livres: do hospital, da UTI, de suporte ventilatório, de suporte cardiovascular e renal - Indicadores de capacidade funcional; força muscular; qualidade de vida; atividade e participação; função pulmonar - Mortalidade até 12 meses da alta hospitalar |

**Quadro 1** - Resumo dos momentos e variáveis de avaliação desde a internação na UTI até 12 meses da alta hospitalar

## PROCEDIMENTOS DE INTERVENÇÃO

Em ambos os grupos (intervenção e controle) os pacientes serão categorizados em níveis funcionais (1-4) através de uma estratégia baseada na escala SOMS ^23^: N_1_ (apresentam movimentação ativa voluntária e são capazes de rolar independentemente, mas não são capazes de transferir de deitado para sentado); N_2_ (são capazes de transferir de deitado para sentado, mas não são capazes de sentar e levantar de uma cadeira); N_3_ (são capazes de sentar e levantar de uma cadeira, mas não são capazes de deambular); N_4_ (são capazes de deambular).

- Grupo cuidados habituais (GCH): receberão mobilização conforme a rotina do serviço: cinesioterapia passiva para pacientes com baixo nível de consciência, incapazes de cooperar e realizarem movimentos ativos voluntários; exercícios ativos e treino de mobilidade para pacientes cooperativos e capazes de realizarem movimentos ativos voluntariamente; eventualmente uso de eletroestimulação e ortostatismo passivo.

Em virtude de ainda não haver um protocolo institucional específico de mobilização, assim como em outros estudos da área ^13^, será realizado um estudo piloto inicial e mensurado o gasto energético diário, o tempo de atividade e o tipo de atividade dos pacientes alocados no grupo cuidados habituais.

- Grupo Intervenção (GI): os pacientes serão submetidos a um protocolo de mobilização progressiva com controle individualizado de dose e carga de treinamento estratificado de acordo com os níveis funcionais e desempenho (Quadro 2). O protocolo será aplicado 1 vez por dia, 5 vezes por semana (segunda a sexta). Os fisioterapeutas do setor realizarão seus cuidados habituais normalmente.

Caso o paciente apresente um desempenho adequado dentro do seu nível funcional e não seja capaz de progredir para um próximo nível, haverá Incremento de volume de treino dentro do próprio nível (Quadro 2). Desempenho adequado dentro do nível funcional significa que o paciente é capaz de completar mais de oito séries ^57^ de exercício e não apresentar aumento da sensação de dor ^16,58^, apresentar sensação de esforço percebido dentro do limite de segurança do protocolo (Borg ≤ 6) ^16,17^ (Escala de 0 a 10) e resposta positiva para o questionamento: “*Você se sente bem depois que faz atividade física*?” ^16,42^.

O treinamento será realizado através de movimentos funcionais progressivos: N_1_ (ponte e rolar para ambos os lados); N_2_ (treino de transferência de deitado para sentado de ambos os lados); N_3_ (sentar e levantar de uma cadeira); N_4_ (treino de marcha). De acordo com seu nível funcional, os pacientes realizarão, 1 vez por dia, (sob supervisão direta dos pesquisadores), 8 series de cada movimento, alternando 20 segundos de execução com 10 segundos de descanso ^57^. Os pacientes serão estimulados constantemente para realizarem os movimentos com a maior velocidade possível e mantendo um Borg máximo de 6 ^17^. Para o treino de sentar e levantar será utilizada uma cadeira hospitalar simples ( largura do assento, profundidade do assento e altura do encosto de 40 cm; altura do assento ao chão de 50 cm).

Os indivíduos poderão progredir de nível ainda que não sejam capazes de executar o movimento independentemente, entretanto, deverão necessitar apenas de assistência mínima ou moderada de no máximo um fisioterapeuta (FSS-ICU ≥ 3) ^46^. Para o treino de sentar e levantar poderá ser utilizada uma cadeira com braços, e para o treino de marcha poderá ser utilizado dispositivos auxiliares (muleta, andador e/ou bengala).

Caso o paciente relate desconforto (Borg > 6) ^17^ ou aumento da dor, será orientado a repousar por até 5 minutos . Não havendo normalização do quadro álgico e da sensação de esforço percebido nesse período de tempo, a sessão de treino será suspensa e realizada após 24 horas.

Uma vez atendido aos critérios de desempenho adequado para aumento de volume de treinamento, serão acrescentados novos exercícios: N_1_ (máxima tríplice flexão/extensão de membros inferiores); N_2_ (rotação/alcance ântero/posterior com a bola); N_3_ (sentar/levantar de uma cadeira jogando a bola na parede e subir/descer degrau); N_4_ (agachar/levantar jogando a bola na parede). Para o exercício de subida e descida de degrau será utilizado uma escada hospitalar (altura do degrau de aproximadamente 39 cm). Para os exercícios com bola, será utilizada uma bola oficial de campo de futebol com aproximadamente 70 cm e 450 g. O anel elástico será da marca [Acte Mini Band Sports.](http://www.netshoes.com.br/produto/kit-acte-mini-band-sports-509-0093-110?campaign=gglepqpla&utm_source=gmc&utm_medium=xml&utm_campaign=gmc_fitness-e-musculacao_tcb-_-xml-_-var_gmc_acte-sports-_-kit-acte-mini-band-sports)

| Treino Inicial | | Incremento de volume de treino |
| --- | --- | --- |
| N_1_ | Ponte, rolar para ambos os lados | Máxima tríplice flexo-extensão alternada de membros inferiores |
| N_2_ | Transferência de deitado para sentado de ambos os lados | Rotação/alcance ântero-posterior com a bola, sentado com os pés apoiados numa escada hospitalar. |
| N_3_ | Sentar/levantar de uma cadeira | Sentar/levantar de uma cadeira jogando a bola na parede (uso de um anel elástico abaixo dos joelhos); subir e descer degrau da escada hospitalar |
| N_4_ | Treino de marcha | Agachar/levantar jogando a bola na parede com uso de um anel elástico abaixo dos joelhos; treino de marcha |

**Quadro 2 –** Protocolo de mobilização progressiva estratificado por níveis funcionais e desempenho.

Antes da primeira realização de um exercício específico do protocolo, os pacientes serão orientados quanto aos aspectos biomecânicos mais adequados. Para tal, serão realizadas demonstrações e realização de até 3 repetições lentas do exercício.

Será realizado um estudo piloto inicial para confirmação do tamanho da amostra, do poder do estudo, e para comparar o gasto energético do grupo intervenção com o grupo controle.

## CRITÉRIOS DE SEGURANÇA

O treinamento será iniciado e interrompido de acordo com os critérios de segurança do Expert consensus and recommendations on safety criteria for active mobilization of mechanically ventilated critically ill adults ^6^. A equipe clínica da UTI poderá interromper a mobilização e os testes funcionais se a condição clínica do paciente se deteriorar. Os pesquisadores envolvidos receberão treinamento específico de acordo com a função que executarão (intervenção ou avaliação) e participarão de reuniões com as coordenações médica, de enfermagem e de fisioterapia do hospital.

O processo de mobilização dos pacientes apresenta riscos mínimos apontados em relatórios nacionais do Departamento de Fisioterapia da Associação de Medicina Intensiva Brasileira ^59^ e internacionais da European Respiratory Society and European Society of Intensive Care Medicine ^14^. Para um controle ainda mais rigoroso, durante todos os procedimentos de avaliação e intervenção haverá fisioterapeutas especialistas na área de fisioterapia em fisioterapia intensiva; serão acrescidos ao Expert consensus and recommendations on safety criteria for active mobilization of mechanically ventilated critically ill adults critérios para interrupção da mobilização dispostos na revisão sistemática publicada na *Cardio pulmonary Physical Therapy Journal* ^11^. Esses critérios (Quadro 3) compõem um fenótipo de segurança para mobilizar pacientes em UTI e estão dispostos em variáveis hemodinâmicas, ventilatórias, de oxigenação, neurológicas, bem como o nível de assistência farmacológica/ventilatória prestada, além de possível queixa referida pelo paciente.

| **Variável** | **Especificação dos critérios** |
| --- | --- |
| *Hemodinâmica* | Queda maior que 20% na frequência cardíaca de repouso; FC < 40 bpm; Episódio de arritmia recente; Introdução recente de drogas antiarrítmicas; IAM recente comprovado por ECG ou enzimas; Queda maior que 20% na PAS ou PAD; Hipotensão Ortostática; PAM < 65 mmhg; novo vasopressor ou escalada da dose do vasopressor. |
| *Ventilação/oxigenação* | FR < 5 ipm ou FR > 40 ipm; Queda de saturação maior que 4%; Saturação < 88-90 %; FIO_2_ ≥ 60 %; PEEP ≥ 10 cmH_2_O; Assincronia paciente e ventilador; Mudança para modo assisto-controlado; Via aérea obstruída. |
| *Neurológicos* | Paciente agitado requerendo sedação ou escalada de sedativos; Intolerância ao esforço e recusa. |
| FCM – Frequência cardíaca máxima; bpm – Batimentos por minuto; IAM – Infarto Agudo do Miocárdio; ECG – Eletrocardiograma; PAS – Pressão Arterial Sistólica; mmhg – Milímetros de Mercúrio; PAD - Pressão Arterial Diastólica; PAM - Pressão Arterial Média; FR – Frequência Respiratória; ipm – Incursões por minuto; FIO_2_ – Fração inspirada de oxigênio; PEEP – Pressão expiratória final positiva em vias aéreas; cmH_2_O – centímetros de água. | |

**Quadro 3** - Critérios para interrupção do protocolo de mobilização. Fonte: Adler e Malone (2012) ^11^.

## DESFECHOS

Primário: Indicadores de custos em saúde

Secundários: Força e massa muscular, FAM-UTI, acelerometria, indicadores de capacidade funcional, qualidade de vida, atividade e participação e função pulmonar, mortalidade e sobrevida em 12 meses.

# TRADUÇÃO, ADAPTAÇÃO TRANSCULTURAL, VALIDAÇÃO E AVALIAÇÃO DAS PROPRIEDADES PSICOMÉTRICAS

Os processos de tradução e adaptação cultural seguirão as seguintes etapas: preparação, tradução, reconciliação, síntese, tradução reversa, revisão, aprovação e pré-teste ^60,61^. Após esses processos, as versões em português dos dois instrumentos serão utilizadas por dois pesquisadores na avaliação de pacientes críticos em UTI seguindo a metodologia de uma recente validação para português de uma escala de avaliação funcional em terapia intensiva ^62^. 1). Preparação: o autor do projeto entrará em contato com os autores da SOMS (Surgical Intensive Care Unit Optimal Mobilization Score) ^63^ e do Expert consensus (Expert consensus and recommendations on safety criteria for active mobilization of mechanically ventilated critically ill adults) ^6^, sendo solicitado os direitos autorais para uso, tradução e validação; 2) Tradução do inglês para o português: a tradução para o português será realizada de forma independente por dois tradutores nativos na língua portuguesa e fluentes na língua inglesa, sendo um familiarizado com os documentos originais do SOMS e do Expert consensus e ciente do objetivo do presente estudo e outro não familiarizado; 3) Reconciliação e síntese: será realizada a comparação entre as duas versões iniciais em português, item a item, com as versões originais por dois fisioterapeutas familiarizados com os documentos originais do SOMS e do Expert consensus. Eventuais discrepâncias existentes serão analisadas e discutidas entre três pesquisadores, levando a síntese de uma segunda versão em português; 4) Tradução reversa: a versão em português será encaminhada para dois tradutores nativos na língua inglesa e fluentes na língua portuguesa para a realização da tradução reversa, sendo que ambos não terão contato com as versões originais em inglês; 5) Revisão e harmonização das traduções reversas: as traduções reversas e versões originais serão comparadas por um comitê revisor composto por três pesquisadores para identificar possíveis discrepâncias entre as versões e realizar os ajustes necessários, item por item, formulando a versão final da tradução reversa; 6) Aprovação dos autores originais: as versões finais traduzidas reversamente para o inglês será enviada aos autores originais para suas avaliações e comentários a respeito de suas consistências. Um comitê de peritos formado por três fisioterapeutas analisará as avaliações e comentários dos autores originais, sendo incorporadas as sugestões e gerando a formulação da última versão em português; e 7) Pré-teste: com a versão final em português, será realizado um treinamento com os avaliadores para o uso. Após o treinamento será realizado um estudo piloto, no qual dois avaliadores aplicarão a SOMS e o Expert consensus seguindo a metodologia descrita nos artigos originais.

Para validação e avaliação das propriedades psicométricas será realizada coleta de dados por dois avaliadores, sendo o avaliador 1 um fisioterapeuta sênior (> 5 anos de experiência) e o avaliador 2 um fisioterapeuta júnior (< 5 anos de experiência). Os dois avaliadores realizarão a pontuação da SOMS e aplicação dos critérios do Expert consensus sem nenhuma comunicação entre si e com fichas separadas ^62^.

# VALIDAÇÃO DE DISPOSITIVO DE BAIXO CUSTO PARA AVALIAÇÃO DE ACELEROMETRIA

No momento da aplicação do protocolo de intervenção serão registrados dados de motricidade capturados de forma digital (através de filmagem) com a finalidade de validar o CvMo*b* ^64^, uma ferramenta de código aberto, disponibilizada gratuitamente (<https://sites.google.com/site/cvmobufba/>) e que é capaz de analisar as trajetórias e determinar as variáveis cinemáticas de um filme obtido por uma câmera simples ^64^.

Os dados de acelerometria obtidos através do software CvMob serão comparados com os dados obtidos pelo ActivPALTM Professional (padrão ouro).

# ANÁLISE ESTATÍSTICA

Para análise dos dados demográficos e clínicos, serão utilizadas estatísticas descritivas. Os dados de variáveis contínuas serão analisados com medidas de tendência central e dispersão. Os dados das variáveis categóricas serão analisados com medidas de frequência.

Para a realização da estatística inferencial serão realizados inicialmente testes para normalidade e de homogeneidade de variância. Desde que os dados se encontrem normalmente distribuídos, o teste *t de student* será utilizado para comparar as diferenças das médias das variáveis, caso contrário, será utilizado o teste Mann-Whitney*.*

A associação da FMA-UTI, variáveis derivadas da acelerometria, mortalidade e sobrevida com as variáveis de interesse (dispostas nos objetivos específicos) será testada por regressão logística múltipla (método de entrada *stepwise forward*). Após o ajuste, permanecerão no modelo final apenas as variáveis que apresentaram p ≤ 0.05.

Para a validação e avaliação das propriedades psicométricas da escala Surgical Intensive Care Unit Optimal Mobilization Score (SOMS) e do Expert consensus and recommendations on safety criteria for active mobilization of mechanically ventilated critically ill adults, será inicialmente verificado o grau de concordância entre os avaliadores utilizando o índice kappa ponderado com intervalo de confiança de IC95%. Para verificar a confiabilidade (consistência interna) das pontuações será utilizado o coeficiente alfa de Cronbach. Além disso, a disposição gráfica de Bland-Altman será utilizada para verificar a concordância da pontuação total entre os avaliadores. As proporções de avaliação com pontuação mínima (piso) e máxima (teto) também serão calculadas. Serão avaliados a normalidade e homocedasticidade para escolha do teste apropriado de coeficiente de correlação. Para avaliação da concordância entre os dados de acelerometria do CvMob e do ActivPALTM Professional será utilizado o coeficiente de StLaurent e o gráfico de Bland-Altman.

O nível de significância estabelecido será de 5% com intervalo de confiança de 95%. A análise estatística será realizada com o uso do software livre SAS University Edition^®^ (SAS Institute Inc., Cary, NC, EUA). Todas as análises do estudo serão realizadas segundo o princípio de intenção de tratar.

# ASPECTOS ÉTICOS

Antes da realização de qualquer procedimento todos os responsáveis legais ou participantes receberão esclarecimentos pormenorizados sobre justificativas, objetivos, hipóteses, procedimentos e intervenções propostas e, então, caso concordem em participar do estudo, deverão assinar um Termo de Consentimento Livre e Esclarecido (ANEXO), previamente elaborado pelos autores do projeto em consonância com as recomendações do CNS, resolução 466/12 CNS/MS ^20^ permitindo a sua participação no estudo. Este projeto será encaminhado ao comitê de ética do Complexo Hospitalar Universitário Professor Edgard Santos da Universidade Federal da Bahia (UFBA), para apreciação, correção e possível aprovação antes da realização da pesquisa.

Medidas de proteção à confidencialidade

As identidades dos participantes serão mantidas em total sigilo por tempo indeterminado tanto pelo executor como pela instituição onde será realizado. Os resultados dos procedimentos executados na pesquisa serão analisados e colocados em tabelas, figuras ou gráficos, podendo ser divulgados em palestras, conferências, periódico científico ou outra forma de divulgação que propicie o repasse dos conhecimentos para a sociedade e para autoridades normativas em saúde nacionais ou internacionais, de acordo com as normas/leis legais regulatórias de proteção nacional ou internacional. Os participantes também receberão uma cópia impressa com o resultado de todos os procedimentos de avaliação bem como informações sobre os resultados da sua avaliação, além dos esclarecimentos necessários.

Critérios para suspender ou encerrar a pesquisa

Em qualquer momento o voluntário poderá obter esclarecimentos sobre todos os procedimentos utilizados na pesquisa e nas formas de divulgação dos resultados.

A participação é voluntária e cada participante tem o direito de se recusar a participar por qualquer razão. Além disso, os participantes poderão desistir em qualquer momento, mesmo após os testes que serão realizados. Está garantida a total autonomia na decisão da participação, bem como na desistência ou retirada da participação no estudo. Se a quantidade de desistências for muito grande o estudo poderá ser interrompido por decisão dos pesquisadores, sendo informado a todos os participantes os motivos que levaram a essa interrupção.

Análise dos riscos e dos benefícios

A pesquisa pode apresentar possíveis desconfortos decorrentes dos testes de desempenho realizados, mas estes são mínimos por se tratarem de medidas utilizadas e já padronizadas na literatura. Os riscos serão ainda minimizados com o acompanhamento constante de um profissional fisioterapeuta especialista durante a realização dos testes. Haverá monitorização permanente dos pacientes durante os procedimentos, avaliando a percepção de esforço, variáveis neurológicas, hemodinâmicas e metabólicas continuamente.

Um dos principais riscos é o de cansaço e/ou fadiga, que será evitado realizando testes em dias diferentes e em momentos diferentes de acordo com percepção de esforço do paciente e critérios de segurança padronizados internacionalmente.

Os resultados obtidos irão colaborar com o conhecimento científico, e direcionar o profissional de saúde para um melhor diagnóstico funcional e uma melhor abordagem terapêutica na população com doença crítica. Os resultados serão divulgados em meios científicos reconhecidos.

# ETAPAS DA PESQUISA E CRONOGRAMA

|  | 2017 | | 2018 | 2019 | 2020 | | 2021 |
| --- | --- | --- | --- | --- | --- | --- | --- |
| ETAPAS/MESES | Julho | Set-Dez | Mar-Dez | Jan-Dez | Jan -Jun | Jul-Dez | Jan-Mar |
| Submissão do Projeto ao CEP | X |  |  |  |  |  |  |
| Aprovação do comitê de ética |  | X |  |  |  |  |  |
| Preparação para coleta de dados |  |  | X |  |  |  |  |
| Estudo piloto |  |  | X |  |  |  |  |
| Recrutamento dos pacientes |  |  | X |  |  |  |  |
| Coleta de Dados |  |  |  | X | X | X |  |
| Analise dos Dados |  |  |  |  |  |  | X |
| Descrição dos Resultados |  |  |  |  |  |  | X |
| Elaboração dos Artigos |  |  |  |  |  |  | X |

Jan = Janeiro; Mar = Março; Abr = Abril; Jun = Junho; Jul=Julho; Set = setembro; Out= Outubro; Dez= Dezembro

# ORÇAMENTO

| Item de Dispêndio* | Descrição | Quantidade | Unitário (R$) | Total (R$) |
| --- | --- | --- | --- | --- |
| Equipamento Nacional | Notebook – para armazenamento dos dados e análise | 1 | 3.800,00 | 3.800,00 |
| Equipamento nacional | Impressora laser para impressão | 1 | 700,00 | 700,00 |
| Equipamento nacional | Tablet para auxílio na coleta | 3 | 500,00 | 1.500,00 |
| Equipamento nacional em desenvolvimento | HD externo para bakup das planilhas de acelerometria | 4 | 100,00 | 400,00 |
| Resma Papel oficio A4 | Papel para impressos de fichas de avaliação, TCLE, análises de dados e fichas de avaliação | 10 | 10,00 | 100,00 |
| Kit toner | Tinta em pó usada para impressão instrumentos de coleta | 01 | 200,00 | 200,00 |
| Faixa elástica | Material de consumo, destinado a realização de exercícios resistidos | 03 | 40,00 | 120.00 |
| Bola | Bola de futebol com dimensões oficiais para realização de exercícios ativos | 03 | 80,00 | 240.00 |
| TOTAL | | | | 7.060,00 |

*Os itens de dispêndio serão custeados pelo próprio pesquisador

| MATERIAIS PERMANENTES  Aquisição | Descrição | Quantidade | Unitário (R$) | Total (R$) |
| --- | --- | --- | --- | --- |
| Ultrassom | Equipamento diagnóstico destinado a avaliação da massa muscular | 01 | 84.600,00 | 84.600,00 |
| Calorímetro | Equipamento destinado a avaliação do consumo de oxigênio | 01 | 60.000 | 60.000 |
| TOTAL | | | | 144.600,00 |

*A massa muscular e o consumo de oxigênio não são variáveis fundamentais de desfecho do projeto, só serão mensuradas caso os equipamentos sejam adquiridos através de edital de financiamento de pesquisa.

| EQUIPAMENTOS PERMANENTES disponíveis | Quantidade | Descrição |
| --- | --- | --- |
| Espirômetro | 01 | Equipamento diagnóstico destinado a avaliação da de fluxos, volumes e capacidade ventilatórias |
| Manuvacuômetro | 01 | Equipamento diagnóstico destinado a avaliação da força muscular respiratória |
| Dinamômetro de preensão manual | 01 | Dispositivo hidráulico de avaliação da força de preensão manual |
| Handheld dinamômetro | 01 | Equipamento manual de avaliação da força muscular |
| Acelerômetro | 10 | Equipamento eletrônico, triaxial, acoplado à um software destinado a avaliação de variáveis acelerométricas do movimento humano |

| EXAMES/CONSULTAS/PROCEDIMENTOS | Descrição | Código | Quantidade | Valor unitário (R$) | Total (R$) |
| --- | --- | --- | --- | --- | --- |
| Diagnóstico cinético funcional | Avaliação cinemática e de parâmetros lineares | 02.11.03.001-5 | 600 | 1,26 | 756,00 |
| Diagnóstico cinético funcional | Avaliação funcional muscular | 02.11.03.007-4 | 900 | 1,26 | 1.134,00 |
| Diagnóstico cinético funcional | Avaliação de função e mecânica respiratória | 02.11.03.004-0 | 200 | 1,26 | 252,00 |
| Diagnóstico cinético funcional | Avaliação de movimento (por imagem) | 02.11.03.006-6 | 600 | 1,26 | 756,00 |
| Procedimentos clínicos | Assistência fisioterapêutica nas disfunções musculo esqueléticas (todas as origens) | 03.02.05.003-5 | 900 | 6,35 | 5.715,00 |
| Diagnóstico por ultrassonografia | Ultrassonografias dos demais sistemas | 02.05.02.006-2 | 300 | 24,20 | 7.260,00 |
| Total (R$) | | | | | 15.873,00 |

1. Fonte: Tabela unificada do SUS (SIGTAP/DATASUS) – disponível em: <http://sigtap.datasus.gov.br/tabela-unificada/app/sec/procedimento/exibir/0211030015/09/2017>
2. EXAMES/CONSULTAS/PROCEDIMENTOS serão financiados com recursos do SUS

# IDENTIFICAÇÃO DA EQUIPE DE PESQUISADORES PARTICIPANTES

- Prof. Dr. **Mansueto Gomes Neto**, Fisioterapeuta e mestre em Ciências da Reabilitação pela UFMG, e doutor em Medicina e Saúde pela UFBA, professor do Instituto de Ciências da Saúde da UFBA. Atuará como coordenador do projeto e pesquisador, sendo responsável pela condução do estudo, supervisão, análise dos dados e redação dos artigos.

- Prof. Dr. **Bruno Prata Martinez**, Fisioterapeuta e especialista em Fisioterapia em Terapia Intensiva e Fisioterapia Respiratória pela ASSOBRAFIR/COFFITO e doutor em Medicina e Saúde pela EBMSP, professor do Instituto de Ciências da Saúde da UFBA. Atuará como vice-coordenador do projeto, responsável pela condução do estudo, supervisão, análise dos dados e redação dos artigos.

- Prof. Ms. **Rodrigo Santos de Queiroz**, Fisioterapeuta, especialista em Terapia Intensiva pela Universidade Castelo Branco, mestre em Ciências da Saúde pela UESB, professor do Departamento de Saúde 1 da UESB. Atuará como pesquisador, sendo responsável condução do estudo, análise dos dados e redação dos artigos.

- Prof. Ms. **Micheli Bernardone Saquetto**, Fisioterapeuta e mestre em Ciências da Saúde pela UESB, doutoranda do PPMS da UFBA, professora do Instituto de Ciências da Saúde da UFBA. Atuará como pesquisadora, atuando no armazenamento dos dados dos procedimentos de avaliação, análise dos dados e redação dos artigos.

- Prof. Esp. **Thiago Queiroz Pires**, Fisioterapeuta, especialista em Fisioterapia Cardiorrespiratória pela Universidade Gama Filho, Especialista em Fisioterapia em Terapia Intensiva pela ASSOBRAFIR/COFFITO, Especialista em Fisiologia do Exercício pela Universidade Estácio de Sá, Fisioterapeuta Intensivista do Hospital das Clínicas de Salvador (EBSERH). Atuará como pesquisador, sendo responsável pela implementação do protocolo de exercícios no grupo intervenção.

- Esp. **Liana Accioly Lins Ferraz Melo**, Fisioterapeuta, especialista em Fisioterapia Pneumofuncional com Ênfase em Terapia Intensiva pela SESAB, UNIME e ISG, Fisioterapeuta Intensivista do Hospital das Clínicas de Salvador (EBSERH). Atuará como pesquisadora, sendo responsável pela implementação do protocolo de exercícios no grupo intervenção.

- Esp. **Juliano dos Santos Batista**, Fisioterapeuta, especialista em Fisioterapia Pneumo-Funcional pela Universidade Castelo Branco, especialista em Fisioterapia em Terapia Intensiva pela ASSOBRAFIR, Fisioterapeuta Intensivista do Hospital das Clínicas de Salvador (EBSERH). Atuará como pesquisador, sendo responsável pela implementação do protocolo de exercícios no grupo intervenção.

- Esp. **Luiz Antônio Moreira Pereira**, Médico, Especialista em Radiologia e Diagnóstico Por Imagem pela Universidade Federal da Bahia. Será responsável pela avaliação ultrassonográfica.

- Esp. **Taís Silva Nascimento**, Fisioterapeuta, especialista em Terapia Intensiva Adulto pela Atualiza - Factiva (2018.1). Auxiliará na coleta de dados.

- **Vitória Passos Rodrigues**, Fisioterapeuta pela Universidade federal da Bahia. Auxiliará na coleta de dados.

- **Beatriz Reis Ferreira**, Fisioterapeuta pela Universidade Estadual do sudoeste da Bahia. Auxiliará na coleta de dados.

- **Bianca Bigogno Reis Cazeta**, Fisioterapeuta pela Universidade federal da Bahia. Auxiliará na coleta de dados.

- **Laíris Trindade Silva de Jesus**, Fisioterapeuta pela Universidade federal da Bahia. Auxiliará na coleta de dados.

- **Larissa da Silva de Souza**, Fisioterapeuta pela Escola Bahiana de Medicina e Saúde Publica. Auxiliará na coleta de dados.

- **Sarah Lima Gomes**, Graduanda em fisioterapia pela Universidade federal da Bahia. Auxiliará na coleta de dados.

- **Juliana Martini Matos Serra**, Fisioterapeuta pela Escola Bahiana de Medicina e Saúde Publica. Especialista em Fisioterapia Hospitalar com Ênfase na Terapia Intensiva pela Faculdade Social da Bahia Auxiliará na coleta de dados.

- **Ana Carolina Conceição Ramos**, Fisioterapeuta pela Escola Bahiana de Medicina e Saúde Publica. Pós Graduação em Fisioterapia em UTI pela ATUALIZA CURSOS em andamento Auxiliará na coleta de dados.

- **Flávia Maria Veloso Silva**, Fisioterapeuta pela União Metropolitana de Educação e Cultura, UNIME. Auxiliará na coleta de dados.

- **Adriana Borges Leite**, Fisioterapeuta pela Universidade Católica do Salvador - UCSAL. Possui título de especialista pela ASSOBRAFIR. Fisioterapeuta Intensivista do Hospital das Clínicas de Salvador (EBSERH). Auxiliará na coleta de dados.

- **Adriele de Sousa França**, Fisioterapeuta pela faculdade Zacarias de Góes (Valença-BA). Pós graduação em Fisioterapia Hospitalar com ênfase em Terapia Intensiva Adulto pela INTERFISIO. Auxiliará na coleta de dados.

- **Alana Rios Garcia Santos**, Terapeuta Ocupacional pela Faculdade Bahiana de Medicina e Saúde Pública. Terapeuta Ocupacional do Hospital das Clínicas de Salvador (EBSERH).

- **Andressa Cruz Gomes dos Santos**, Fisioterapeuta graduanda pela Universidade Federal da Bahia (UFBA). Auxiliará na coleta de dados.

- **Luís Artur Santiago dos Santos**, Fisioterapeuta pela Universidade Federal da Bahia (UFBA). Pós Graduação em Fisioterapia Hospitalar com ênfase em UTI pela Escola Bahiana de Medicina e Saúde Pública, em andamento. Auxiliará na coleta de dados.

- **Nara Cristine de Moraes Bonfim**, Fisioterapeuta pela Escola Bahiana de Medicina e Saúde Pública. Pós graduação em Fisioterapia em UTI pela ATUALIZA Cursos, em andamento. Auxiliará na coleta de dados.

- **Tiago Bastos Cardoso**, Fisioterapeuta pelo Centro Universitário da Bahia (FIB). Possui título de especialista pela ASSOBRAFIR. Fisioterapeuta Intensivista do Hospital das Clínicas de Salvador (EBSERH). Auxiliará na coleta de dados.

- **Virgínia Eugênia Pinheiro e Silva**, Fisioterapeuta pela Universidade Católica do Salvador (UCSAL). Fisioterapeuta Intensivista do Hospital das Clínicas de Salvador (EBSERH). Auxiliará na coleta de dados.

- **Marcus Vinicius das Neves**, Fisioterapeuta pela União Metropolitana de Educação e Cultura - UNIME. Possui título de especialista pela ASSOBRAFIR. Auxiliará na coleta de dados.

# DISPONIBILIDADE EFETIVA DE INFRA-ESTRUTURA E DE APOIO TÉCNICO PARA O DESENVOLVIMENTO DO PROJETO

O Hospital Universitário Prof. Edgar Santos (HUPES) possui áreas assistenciais e perfil de pacientes que se adequam ao presente projeto de pesquisa. A ampla maioria de exames e monitorização multiparamétrica necessária para o estudo já é realizada na rotina de serviço do Hospital.

A equipe do estudo possui ampla expertise para condução dos procedimentos de intervenção, proporcionando aporte técnico necessário ao desenho do projeto e reforça a qualidade dos procedimentos planejados.

# REFERÊNCIAS

1. Kelly FE, Fong K, Hirsch N, Nolan JP. Intensive care medicine is 60 years old: the history and future of the intensive care unit. *Clin Med*. 2014;14(4):376-379. doi:10.7861/clinmedicine.14-4-376.

2. Herridge et al. Functional Disability 5 Years after Acute Respiratory Distress Syndrome Margaret. *New Engl J*. 2011;364(14):2545-2559. doi:10.1056/NEJMoa1402685.

3. Schweickert WD, Hall J. ICU-Acquired Weakness. *Chest*. 2007;131(5):1541-1549. doi:10.1378/chest.06-2065.

4. De Jonghe B, Sharshar T, Lefaucheur J, et al. Paresis Acquired in the Intensive Care Unit. *Jama*. 2002;288(22):2859-2867. doi:10.1001/jama.288.22.2859.

5. Skinner EH, Berney S, Warrillow S, Denehy L. Rehabilitation and exercise prescription in Australian intensive care units. *Physiotherapy*. 2008;94(3):220-229. doi:10.1016/j.physio.2007.11.004.

6. Hodgson CL, Stiller K, Needham DM, et al. Expert consensus and recommendations on safety criteria for active mobilization of mechanically ventilated critically ill adults. *Crit Care*. 2014;18(6):1-9. doi:10.1186/s13054-014-0658-y.

7. Stiller K. Physiotherapy in intensive care: Towards an evidence-based practice. *Chest*. 2000;118(6):1801-1813. doi:10.1378/chest.118.6.1801.

8. Stiller K. Physiotherapy in intensive care: An updated systematic review. *Chest*. 2013;144(3):825-847. doi:10.1378/chest.12-2930.

9. Stiller K, Phillips AC, Lambert P. The safety of mobilisation and its effect on haemodynamic and respiratory status of intensive care patients. *Physiother Theory Pract*. 2004;20(3):175-185. doi:10.1080/09593980490487474.

10. Zafiropoulos B, Alison JA, Mccarren B. Physiological responses to the early mobilisation of the intubated , ventilated abdominal surgery patient. *Aust J Physiother*. 2004;50(2):95-100.

11. Adler J. Early Mobilization in the Intensive Care Unit : *Cardiopulm Phys Ther J*. 2012;23(1):5-13.

12. Lee SMC, Moore AD, Everett ME, Stenger MB, Platts SH. Aerobic exercise deconditioning and countermeasures during bed rest. *Aviat Sp Environ Med*. 2010;81(1):52-63. doi:10.3357/ASEM.2474.2010.

13. Castro-Avila AC, Serón P, Fan E, Gaete M, Mickan S. Effect of early rehabilitation during intensive care unit stay on functional status: Systematic review and meta-analysis. *PLoS One*. 2015;10(7):1-21. doi:10.1371/journal.pone.0130722.

14. Gosselink R, Bott J, Johnson M, et al. Physiotherapy for adult patients with critical illness: Recommendations of the European Respiratory Society and European Society of Intensive Care Medicine Task Force on Physiotherapy for Critically Ill Patients. *Intensive Care Med*. 2008;34(7):1188-1199. doi:10.1007/s00134-008-1026-7.

15. Morris PE, Goad A, Thompson C, et al. Early intensive care unit mobility therapy in the treatment of acute respiratory failure. *Crit Care Med*. 2008;36(8):1-6. doi:10.1097/CCM.0b013e318180b90e.

16. Hickmann CE, Zapatero DC, Bialais E, et al. Teamwork enables high level of early mobilization in critically ill patients. *Ann Intensive Care*. 2016;6(1):80. doi:10.1186/s13613-016-0184-y.

17. Borg G, Ljunggren G, Ceci R. The increase of perceived exertion, aches and pain in the legs, heart rate and blood lactate during exercise on a bicycle ergometer. *Eur J Appl Physiol Occup Physiol*. 1985;54(4):343-349. doi:10.1007/BF02337176.

18. Moher D, Hopewell S, Schulz KF, et al. CONSORT 2010 explanation and elaboration: Updated guidelines for reporting parallel group randomised trials. *Int J Surg*. 2012;10(1):28-55. doi:10.1016/j.ijsu.2011.10.001.

19. Verceles A. Use of Accelerometry to Monitor Physical Activity in Critically Ill Subjects: A Systematic Review. *Respir Care*. 2015;60(9):1330-1336. doi:10.4187/respcare.03677.Use.

20. Conselho Nacional de Saúde (Brasil). Resolução N^o^ 466, de 12 DE Dezembro de 2012. 2012. http://conselho.saude.gov.br/resolucoes/2012/Reso466.pdf.

21. Barros LDS. Agência Nacional de Vigilância Sanitária (Programa Nacional De Prevenção e Controle de Infecções Relacionadas à Assistência à Saúde). 2016.

22. Mahoney FI, Barthel DW. Functional evaluation: the barthel index. *Md State Med J*. 1965;14:61-65. http://www.ncbi.nlm.nih.gov/pubmed/14258950. Accessed April 11, 2017.

23. Schaller SJ, Anstey M, Blobner M, et al. Early, goal-directed mobilisation in the surgical intensive care unit: a randomised controlled trial. *Lancet*. 2016;388(10052):1377-1388. doi:10.1016/S0140-6736(16)31637-3.

24. Altman DG, Bland JM. How to randomise. *BMJ Br Med J*. 1999;319(7211):703-704. doi:10.1136/bmj.319.7211.703.

25. Randelli P, Arrigoni P, Lubowitz JH, Cabitza P, Denti M. Randomization Procedures in Orthopaedic Trials. *Arthrosc - J Arthrosc Relat Surg*. 2008;24(7):834-838. doi:10.1016/j.arthro.2008.01.011.

26. Kim J, Shin W. How to do random allocation (randomization). *Clin Orthop Surg*. 2014;6(1):103-109. doi:10.4055/cios.2014.6.1.103.

27. Suresh K. An Overview of Randomization Techniques: An Unbiased Assessment of Outcome in Clinical Research. *J Hum Reprod Sci*. 2011;4(1):8-11. doi:10.4103/0974-1208.82352.

28. Doig GS, Simpson F. Randomization and allocation concealment: A practical guide for researchers. *J Crit Care*. 2005;20(2):187-191. doi:10.1016/j.jcrc.2005.04.005.

29. Corcoran JR, Herbsman JM, Bushnik T, et al. Early Rehabilitation in the Medical and Surgical Intensive Care Units for Patients With and Without Mechanical Ventilation: An Interprofessional Performance Improvement Project. *Pm&R*. 2017;9:1-7. doi:10.1016/j.pmrj.2016.06.015.

30. YOUNG, Paul et al. End points for phase II trials in intensive care: recommendations from the Australian and New Zealand Clinical Trials Group consensus panel meeting. *Crit Care Resusc*. 2012;14(13):211.

31. Kleyweg RP, van der Meché FG, Schmitz PI. Interobserver agreement in the assessment of muscle strength and functional abilities in Guillain-Barré syndrome. *Muscle Nerve*. 1991;14(11):1103-1109. doi:10.1002/mus.880141111.

32. Hermans G, Clerckx B, Vanhullebusch T, et al. Interobserver agreement of Medical Research Council sum-score and handgrip strength in the intensive care unit. *Muscle Nerve*. 2011;45(1):18-25. doi:10.1002/mus.22219.

33. Vanpee G, Hermans G, Segers J, Gosselink R. Assessment of limb muscle strength in critically ill patients: a systematic review. *Crit Care Med*. 2014;42(3):701-711. doi:10.1097/CCM.0000000000000030.

34. Ali NA, O’Brien JM, Hoffmann SP, et al. Acquired weakness, handgrip strength, and mortality in critically III patients. *Am J Respir Crit Care Med*. 2008;178(3):261-268. doi:10.1164/rccm.200712-1829OC.

35. Baldwin CE, Paratz JD, Bersten AD. Muscle strength assessment in critically ill patients with handheld dynamometry: An investigation of reliability, minimal detectable change, and time to peak force generation. *J Crit Care*. 2013;28(1):77-86. doi:10.1016/j.jcrc.2012.03.001.

36. Samosawala NR, Vaishali K, Kalyana BC. Measurement of muscle strength with handheld dynamometer in Intensive Care Unit. *Indian J Crit Care Med*. 2016;20(1):21-26. doi:10.4103/0972-5229.173683.

37. Guralnik JM, Simonsick EM, Ferrucci L, et al. A Short Physical Performance Battery Assessing Lower Extremity Function : Association With Self-Reported Disability and Prediction of Mortality and Nursing Home Admission. 1994;49(2):85-94.

38. Hermans G, Van den Berghe G. Clinical review: intensive care unit acquired weakness. *Crit Care*. 2015;19(1):274. doi:10.1186/s13054-015-0993-7.

39. Puthucheary Z a, Rawal J, McPhail M, et al. Acute Skeletal Muscle Wasting in Critical Illness. *J Am Med Assoc*. 2013;310(15):1-10. doi:10.1001/jama.2013.278481.

40. Seymour JM, Ward K, Sidhu PS, et al. Ultrasound measurement of rectus femoris cross-sectional area and the relationship with quadriceps strength in COPD. *Thorax*. 2009;64(5):418-423. doi:10.1136/thx.2008.103986.

41. Kress JP, Hall JB, Trial S profile of extended-release niacin in the A-H. ICU-Acquired Weakness and Recovery from Critical Illness. *N Engl J Med*. 2014;371(3):288-290. doi:10.1056/NEJMc1406274.

42. Beach L, Fetterplace K, Edbrooke L, et al. Measurement of physical activity levels in the Intensive Care Unit and functional outcomes: An observational study. *J Crit Care*. 2017;40:189-196. doi:10.1016/j.jcrc.2017.04.006.

43. Demeyer H, Burtin C, Van Remoortel H, et al. Standardizing the analysis of physical activity in patients with COPD following a pulmonary rehabilitation program. *Chest*. 2014;146(2):318-327. doi:10.1378/chest.13-1968.

44. Godfrey A, Conway R, Leonard M, Meagher D, Ólaighin GM. Motion analysis in delirium: A discrete approach in determining physical activity for the purpose of delirium motoric subtyping. *Med Eng Phys*. 2010;32(2):101-110. doi:10.1016/j.medengphy.2009.10.012.

45. Meyer M, Stanislaus A, Lee J, Waak K. Surgical Intensive Care Unit Optimal Mobilisation Score (SOMS) trial: a protocol for an international, multicentre, randomised controlled trial focused on goal-directed. *BMJ Open*. 2013;3(e003262):1-11. doi:10.1136/bmjopen-2013-003262.

46. Thrush A, Rozek M, Dekerlegand JL. The clinical utility of the functional status score for the intensive care unit (FSS-ICU) at a long-term acute care hospital: a prospective cohort study. *Phys Ther*. 2012;92(12):1536-1545. doi:10.2522/ptj.20110412.

47. Jones J, Rikli R. Measuring Functional. *J Act Aging*. 2002;(April):24-30. doi:10.1016/j.neuroimage.2011.02.054.

48. Crapo RO, Casaburi R, Coates AL, et al. ATS statement: Guidelines for the six-minute walk test. *Am J Respir Crit Care Med*. 2002;166(1):111-117. doi:10.1164/rccm.166/1/111.

49. Enrigth. Reference Equations for the Six-Minute Walk in Healthy Adults. *Am J Respir Crit Care Med*. 1998;161(4):1396-1396. doi:10.1164/ajrccm.161.4.16147a.

50. Ciconelli, RM et al. Tradução para a língua portuguesa e validação do questionário genérico de avaliação de qualidade de vida SF-36 (Brasil-SF36). *Rev bras Reum*. 1999;39:143-150.

51. Silveira C, Angela M, Carvalho R, et al. Adaptação transcultural da Escala de Avaliação de Incapacidades da Organização Mundial de Saúde (WHODAS 2.0) para o Português. *Rev Assoc Med Bras*. 2013;59(3):234-240.

52. Marson F, Martins MA, Coletto FA, Campos AD. Correlação entre o Consumo de Oxigênio Obtido pelo Método de Fick e pela Calorimetria Indireta no Paciente Grave. 2003;82(n^o^ 1):72-76.

53. Reeves MM, Davies PSW, Bauer J, et al. Reducing the time period of steady state does not affect the accuracy of energy expenditure measurements by indirect calorimetry. 2004:130-134.

54. Gibson GJ, Whitelaw W, Siafakas N, et al. ATS/ERS Statement on respiratory muscle testing. *Am J Respir Crit Care Med*. 2002;166(4):518-624. doi:10.1164/rccm.166.4.518.

55. SBPT. SOCIEDADE BRASILEIRA DE PNEUMOLOGIA E TISIOLOGIA. Diretrizes para testes de função pulmonar. *J Pneumol*. 2002;28(Suppl 3):S1-S238.

56. Miller MR, Hankinson J, Brusasco V, et al. Standardisation of spirometry. *Eur Respir J*. 2005;26(2):319-338. doi:10.1183/09031936.05.00034805.

57. Tabata I, Nishimura K, Kouzaki M, et al. Effects of moderate-intensity endurance and high-intensity intermittent training on anaerobic capacity and VO2max. *Med Sci Sport Exerc*. 1996;28(10):1327-1330. doi:10.1097/00005768-199610000-00018.

58. Sousa FF, Pereira LV, Cardoso R, Hortense P. Escala Multidimensional de Avaliação de Dor ( EMADOR ). *Rev Latino-am Enferm*. 2010;18(1):9. doi:10.1590/S0104-11692010000100002.

59. Cavalcanti R, Duarte A, Prata B, Aquim EE. Fisioterapia em pacientes críticos adultos : recomendações do Departamento de Fisioterapia da Associação de Medicina Intensiva Brasileira. 2012;24(1):6-22.

60. Beaton DE, Bombardier C, Guillemin F, Ferraz MB. Guidelines for the Process of Cross-Cultural Adaptation of Self-Report Measures. 2000;25(24):3186-3191.

61. Wild D, Grove A, Martin M, et al. Principles of Good Practice for the Translation and Cultural Adaptation Process for Patient-Reported Outcomes ( PRO ) Measures : Report of the ISPOR Task Force for Translation and Cultural Adaptation. 2005;8(2):94-104.

62. Maria Y, Kawaguchi F, Nawa RK, Figueiredo TB, Martins L, Pires-neto RC. Perme Intensive Care Unit Mobility Score e ICU Mobility Scale : tradução e adaptação cultural para a língua portuguesa falada no Brasil. 2016;42(6):429-434.

63. Garzon-serrano J, Ryan C, Waak K, et al. Early Mobilization in Critically Ill Patients : Patients ’ Mobilization Level Depends on Health Care Provider ’ s Profession. *PMRJ*. 2011;3(4):307-313. doi:10.1016/j.pmrj.2010.12.022.

64. Pena N, Credidio BC, Corrêa LPNRMS, França, Lucas Gabriel Souza Cunha, Marcelo do Vale Sousa MC de, Vieira JPBC, Miranda JGV. Instrumento livre para medidas de movimento. *Rev Bras Ensino Física*. 2013;35(3):3505-3509. doi:10.1590/S1806-11172013000300024.

# APÊNDICE

**INSTRUMENTO DE COLETA DE DADOS**

| Nome do paciente: | | | N° ( ) |
| --- | --- | --- | --- |
| Nome do Responsável: | | | ^Grau de parentesco^ |
| Data da coleta de dados inicial: / / | | Horário: _(h)_ : _(min)_ | |
| Endereço - Rua: | Bairro: | | Nº |
| Cidade: | | Telefone: | |
| Outras formas de contato:  Telefone (2)______________Telefone (3)_____________Telefone(4)______________  e-mail ______________________________ rede social_________________________ | | | |

1. Sexo: Masc ( ) Fem ( ) 2. Idade: ________ 3. Escolaridade: Analfabeto ( ) Alfabetizado ( ) Grau de instrução: ___________4. Estado Civil: Solteiro(a) ( ) Casado(a)/juntado(a) ( ) Divorciado(a) ( ) Viúvo(a) ( ) 5. Trabalha: Sim ( ) Não ( ) Em que? _______________________________ 6. Se não trabalha: é aposentado? Sim ( ) Não ( )

7. Qual a atividade que exercia? _____________________________________________

8. Possuia alguma doença antes da internação? Sim ( ) Não ( ) Se sim. Quais?__________________________________________________________________

9. BARTHEL _(recordtório duas semanas antes da admissão na UTI)_ _______________

**Dados do prontuário**

1. Motivo da internação na UTI:

1º_________________________________________ [CID:](about:blank) ____________

2º_________________________________________ CID: ____________

2. Problemas clínicos adicionais:

1º_________________________________________ [CID:](about:blank) ____________

2º_________________________________________ CID: ____________

3º_________________________________________ CID: ____________

3. Diagnóstico de sepse (não) (sim) 4. Diagnóstico de SDRA (não) (sim)

5. Data da internação no hospital _____/_______/________ 6. Horário da internação no hospital ( : ) 7. Ventilação mecânica antes da UTI (não) (sim) *se sim* data _____/_______/________ e Horário ( : )

8. APACHE _______________ _(dados coletados nas primeiras 24h)_

| Temperatura(ºC): | Sódio(mMol/L): |
| --- | --- |
| Pressão Arterial Média (mmHg): | Potássio(mMol/L): |
| Frequência Cardíaca (bpm): | Creatinina(mg/100ml): |
| Frequência Respiratória (ipm): | Hematócrito (%): |
| Oxigenação (PaO_2_/FiO_2_): | Glóbulos Brancos(total/mm^3^): |
| pH Arterial (nmoles/L): | Escala de Coma de Glasgow: |

**Dados para composição do Apache II e mortalidade estimada (comorbidades)**

***Cardiovascular***

( ) Insuficiência Cardíaca Congestiva (ICC)

( ) Infarto Agudo do Miocárdio (IAM)

( ) Hipertensão Arterial Sistêmica (HAS)

( ) Doença Arterial Periférica (DAP)

( ) Trombose venosa profunda (TVP)

( ) Dislipidemia

( ) Outras: ___________________________________________________________

***Pulmonar***

( ) Doença Pulmonar Obstrutiva Crônica (DPOC)

( ) Hipertensão Pulmonar (HP)

( ) Dependência de Oxigênio

( ) Tromboembolismo Pulmonar (TEP)

( ) Outras: ___________________________________________________________

***Renal-Metabólica***

( ) Insuficiência Renal Crônicanão-dialítica

( ) Insuficiência Renal Crônica Dialítica

( ) Obesidade

( ) Diabetes mellitus (DM)

( ) Outras: ___________________________________________________________

***Hepática***

( ) Cirrose por ______________________

( ) Encefalopatia

( ) Hemorragia Digestiva Alta

( ) Hemorragia Digestiva Baixa

***Neurológica***

( ) Acidente Vascular Encefálico (AVE)

( ) Demência

( ) Epilepsia

***CirurgiaPrévia:*** ______________________ 9. Mortalidade estimada (____________)

10. Dados antropométricos: Peso __________________ Altura ______________________

| **(Ficha 1) - Planilha de coleta de dados diária durante a internação** | | | | | | | | | | | | |
| --- | --- | --- | --- | --- | --- | --- | --- | --- | --- | --- | --- | --- |
| **Variáveis / UTI** | D1 | | | D2 | | | D3 | | | Dn | | |
| Data | / / | | | / / | | | / / | | | / / | | |
| Sedação / Bloqueio |  | | |  | | |  | | |  | | |
| Glasgow/Ramsay |  | | |  | | |  | | |  | | |
| Neurológicos |  | | |  | | |  | | |  | | |
| Ventilação/oxigenação |  | | |  | | |  | | |  | | |
| Hemodinâmica |  | | |  | | |  | | |  | | |
| Corticoide | (Sim) (Não) | | | (Sim) (Não) | | | (Sim) (Não) | | | (Sim) (Não) | | |
| Glicemia _(maior valor)_ |  | | |  | | |  | | |  | | |
| Tipo de Nutrição |  | | |  | | |  | | |  | | |
| Sinais de Infecção | (Sim) (Não) | | | (Sim) (Não) | | | (Sim) (Não) | | | (Sim) (Não) | | |
| Sinais de inflamação | (Sim) (Não) | | | (Sim) (Não) | | | (Sim) (Não) | | | (Sim) (Não) | | |
| Antibiótico | (Sim) (Não) | | | (Sim) (Não) | | | (Sim) (Não) | | | (Sim) (Não) | | |
| Mobilização | (Sim) (Não) | | | (Sim) (Não) | | | (Sim) (Não) | | | (Sim) (Não) | | |
| SOMS atingido |  | | |  | | |  | | |  | | |
| Capacidade Interação | (Sim) (Não) | | | (Sim) (Não) | | | (Sim) (Não) | | | (Sim) (Não) | | |
| FSS_/Rolar/Sup-Sedst/Seds.SA_ |  |  |  |  |  |  |  |  |  |  |  |  |
| FSS_/Seds.Ortostase/Deambula_ |  |  |  |  |  |  |  |  |  |  |  |  |
| Mobilização | (Sim) (Não) | | | (Sim) (Não) | | | (Sim) (Não) | | | (Sim) (Não) | | |
| FL Mob indep. |  | | |  | | |  | | |  | | |
| **Códigos para registro de alterações nas últimas 24 h** | | | | | | | | | | | | |
| **Sedação / Bloqueio =** 0 = nenhum; 1 = sedação; 2 = bloqueio | | | | | | | | | | | | |
| **Neurológicos:** 0 = nenhum; 1 = agitação; 2 = escalada de sedativos; 3 = Intolerância ao esforço; 4 = recusa. | | | | | | | | | | | | |
| **Ventilação/oxigenação:** 0 = nenhum; 1 = FR < 5 ipm; 2 = FR > 40 ipm; 3 = Queda de Spo2 >4%; 4 = Saturação < 90%; 5 = FIO2 ≥ 60 %; 6 = PEEP ≥ 10 cmH2O; 7 = Assincronia paciente e ventilador;8 = Mudança para modo assisto-controlado; 9= Via aérea obstruída. | | | | | | | | | | | | |
| **Hemodinâmica:** 0 = nenhum; 1 = Queda > 20%; 2 = FCrepouso; 3 = FC < 40 bpm; 4 = Episódio de arritmia recente; 5 = Introdução recente de drogas antiarrítmicas; 6 = IAM recente comprovado por ECG ou enzimas; 7 = Queda > 20% na PAS ou PAD; 8 = Hipotensão Ortostática; 9= PAM < 65 mmhg; 10 = novo vasopressor ou escalada da dose do vasopressor. | | | | | | | | | | | | |
| **Tipo de Nutrição:** 0 = nenhum; 1 = Oral; 2 = Parenteral; 3 = Enteral | | | | | | | | | | | | |
| **Sinais de Infecção e Sinais de inflamação =** confirmar através do médico plantonista | | | | | | | | | | | | |
| **FL Mob indep.** **=** Fatores limitantes da mobilização independente do paciente: 0 = nenhum; 1 = cirurgia, 2 = transferência; 3 = procedimentos de imagem; 4 = procedimentos de enfermagem; 5 = outro (especificar) | | | | | | | | | | | | |

11. Força Muscular (Avaliação de 3 em 3 dias)

|  | D1 | | | D2 | | | D3 | | | Dn | | |
| --- | --- | --- | --- | --- | --- | --- | --- | --- | --- | --- | --- | --- |
| Data | / / | | | / / | | | / / | | | / / | | |
| MRC_MSdireito_ |  |  |  |  |  |  |  |  |  |  |  |  |
| MRC_MSesquerdo_ |  |  |  |  |  |  |  |  |  |  |  |  |
| MRC_MIdireito_ |  |  |  |  |  |  |  |  |  |  |  |  |
| Handheld_MSdireito_ |  |  |  |  |  |  |  |  |  |  |  |  |
| Handheld_MSesquerdo_ |  |  |  |  |  |  |  |  |  |  |  |  |
| Handheld_MIdireito_ |  |  |  |  |  |  |  |  |  |  |  |  |
| Handheld_MIesquerdo_ |  |  |  |  |  |  |  |  |  |  |  |  |
| F. de preensão |  |  |  |  |  |  |  |  |  |  |  |  |
| Teste de sentar e levantar |  |  |  |  |  |  |  |  |  |  |  |  |

12. Acelerometria

| Data | D1 | D2 | D3 | D4 | D5 | Dn |
| --- | --- | --- | --- | --- | --- | --- |
|  | / / | / / | / / | / / | / / | / / |
| Gasto energético |  |  |  |  |  |  |
| Número diário de passos |  |  |  |  |  |  |
| Tempo de atividade e repouso |  |  |  |  |  |  |
| Tempo gasto em atividade física |  |  |  |  |  |  |
| Equivalentes metabólicos médios |  |  |  |  |  |  |
| Tempo gasto na posição deitada |  |  |  |  |  |  |
| Tempo gasto na posição sentada |  |  |  |  |  |  |
| Número de transições diárias |  |  |  |  |  |  |

13. Tempo de início da mobilização (h) _______________

14. Massa muscular _em até 48h_ (________) Data _____/_______/________ Horário ( : )

15. Nutrição: Data de início _____/_______/________ Horário ( : )

*introduzida nas primeiras 24 h ( ) ou entre 24-48 h da admissão ( )*

16. Desmame:

( ) Simples (1 tentativa de TRE) ( ) Difícil (até 7 dias do 1ª TRE) ( ) Prolongado (mais de 7 dias após a 1ª TRE)

17. Extubação ( ) Sucesso ( ) Falha Nº de falhas ( )

18. Uso de TQT ( ) sim ( ) não

19. Uso de VNI ( ) sim ( ) não

20. Desfecho UTI

Data da Alta da UTI _____/_____/_____ Horário:___________

Data do Óbito _____/_____/_____ (Utilizar somente se o paciente falecer na UTI)

Informações relevantes da UTI:

________________________________________________________________________________________________________________________________________________________________________________________________________________________

| **(Ficha 2) - Avaliação específicas no dia da alta da UTI** | | | |
| --- | --- | --- | --- |
| Timed Up and Go test |  | | |
| Massa muscular |  |  |  |
| Tempo de prontidão para alta da UTI |  | | |
| Tempo de internação na UTI |  | | |

20. Tempo de internação hospitalar _______________

21. Infecção hospitalar _______________

| **(Ficha 3) - Avaliação (imediatamente antes da alta hospitalar e após alta hospitalar** | | | |
| --- | --- | --- | --- |
| Timed Up and Go test |  | | |
| Massa muscular |  |  |  |
| Peso (Kg) |  |  |  |
| TC6 |  | | |
| Tempo de suporte ventilatório |  | | |
| Tempo de suporte cardiovascular |  | | |
| Tempo de suporte renal |  | | |
| Tempo de reabilitação após a alta hospitalar |  | | |
| Dias livres de hospital |  | | |
| Qualidade de vida |  | | |
| Atividade e participação |  | | |
| Função pulmonar | Anexar relatório da espirometria e manovacuometria | | |
| Indicadores de capacidade funcional |  | | |
| Força muscular |  | | |
